# Circularity Policy Interventions in One Sector Can Reduce Circularity in Others

**DOI:** 10.1021/acs.est.6c05815

**Published:** 2026-07-22

**Authors:** Mattia Maeder, Dominik Reichert, Magnus Fröhling

**Affiliations:** † Chair of Circular Economy and Sustainability Assessment, 9184 Technical University of Munich (TUM), TUM Campus Straubing for Biotechnology and Sustainability, Am Essigberg 3, 94315 Straubing, Germany

**Keywords:** Recycling, Material Flow Analysis, Policy Analysis, Automotive Industry, Public Policy, Recycled Content Quota, Recycled Content Target, Recycled Content Mandate

## Abstract

Circular economy (CE) policies, such as recycled content standards, target closed-loop circularity and sustainable development. However, few prospective assessments of their impact exist, and it is unclear how circularity policy interventions in one sector may affect circularity in another sector. Here, we assess conditions for increasing closed-loop steel circularity with a newly developed sector-coupled dynamic material flow analysis model of the automotive and building sectors in the EU. We show that improving the scrap quality from end-of-life vehicles is essential to increasing automotive closed-loop circularity until 2050. This improvement inhibits scrap flows to the building sector, reducing its open-loop circularity potential, but alleviates the risk of low-quality, potentially unusable scrap surpluses. Navigating these trade-offs means that CE policy interventions must consider circularity not only within but also across sectors. Overall, all sectors must contribute to preserving the value of materials over multiple life cycles. Beyond insights for policymakers, we provide guidance for industry to increase product circularity and for academia to consider systemic effects in scientific methods.

## Graphical abstract

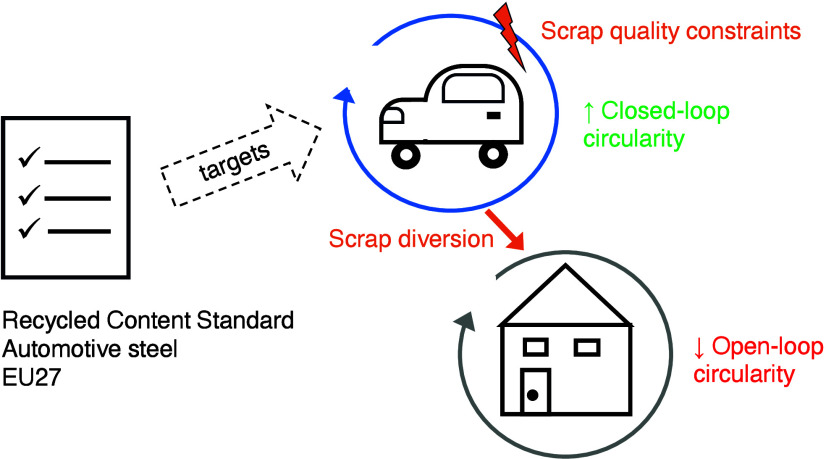

## 1 Introduction

The Circular Economy (CE) offers an alternative to the linear “make-use-dispose” paradigm.^1^ By narrowing, slowing, and closing material cycles in a regenerative system, the goal is to achieve sustainable development.^2^ Despite a growing body of academic research and awareness among companies and policymakers, progress toward CE has been slow. For instance, the Circular Material Use Rate (CMUR) in the European Union was still relatively low at 12.2% in 2024 ^3^ – compared to the target of 24% in 2030 planned in the upcoming Circular Economy Act.^4^

EU policymakers have identified CE as a strategic lever for sustainability and introduced multiple policy instruments,^5^ including extended producer responsibility schemes, eco-design requirements, and recycled content standards (RCSs). RCSs, sometimes also termed recycled content targets, quotas, or mandates, require a minimum recycled content share in new products, often derived from post-consumer waste. They have a transformative character: By targeting material inputs, they go beyond established end-of-life recycling regulations and seek to promote circularity.^6^

RCS examples in the EU include the Battery Regulation, Packaging and Packaging Waste Regulation, and the Proposal for an End-of-Life Vehicle (ELV) Regulation.^7^ The ELV Regulation Proposal includes an RCS for new vehicles. The recycled content should stem not only from post-consumer waste but also partially from ELVs. Thus, this RCS aims to effectively close the loop in the automotive sector. The current Proposal already includes concrete RCS target values for plastics and considers introducing RCSs for metals, including steel, aluminum, magnesium, and rare earth. For steel, the Commission may introduce RCS values through Delegated Acts – subject to technical feasibility studies on material supply and demand, economics, and alignment with climate, environmental, and geopolitical goals.

RCSs for automotive steel could be particularly interesting for multiple reasons. First, compared to automotive lithium-ion battery materials, for which large-scale recycling infrastructure is in its infancy,^8^ and automotive plastics, for which incineration is still common due to the limits of mechanical recycling and the still nascent chemical recycling,^9,10^ the case for steel RCSs is not as intuitive: steel has an established recycling infrastructure. However, while recycling rates are already high, recycled content levels in new cars are low. Automotive steel has stringent quality requirements that are currently challenging to meet using scrap from post-consumer waste. Scrap is contaminated with tramp elements, typically copper,^11^ leading to downcycling, i.e., a loss in quality when materials are reprocessed from waste compared to their original quality. Hence, steel from ELVs usually ends up in the construction sector, which has laxer quality requirements.^12–14^ Second, steel accounts for the largest share by weight in a car, and the automotive industry is the EU’s second-largest consumer of steel.^15^ Concurrently, steel is responsible for around 5% of CO_2_ emissions in the EU. Thus, improving the circularity of automotive steel as envisioned by an RCS may have significant sustainability benefits by increasing recycled content, potentially reducing virgin material consumption and its associated environmental impacts, including greenhouse gas emissions, and alleviating supply chain and geopolitical risks.

Despite RCSs’ potential benefits, ex-ante assessments of their feasibility and implications are scarce. Previous studies have assessed RCSs of battery materials^16,17^ and automotive plastics in the EU.^18^ On steel circularity more broadly, a study for Japan by Nakamura et al. (2014) indicated that 7–8% of recovered steel can be recycled back into the automotive sector,^19^ and Watari et al. (2023) found that scrap-based steel in Japan is limited to 20% of the total steel used for automobiles.^14^ Furthermore, the authors highlight that preserving the value of materials and avoiding downcycling are crucial to achieving CE goals.^14^ However, CE measures and policies should avoid unintended consequences and strive for system-wide benefits – not just individual materials, products, sectors, or countries.^6^ Researchers have identified risks like regional leakage effects, where high steel circularity in one region may be offset by low circularity elsewhere due to trade patterns,^20^ and market displacement effects, where improving closed-loop circularity for packaging plastics may not reduce primary plastics consumption overall.^21,22^

Thus, the current academic debate centers on downcycling and underscores the need for system-wide benefits when implementing CE policies. Studies on the automotive industry link the influence of ELV regulation to circularity outcomes.^23^ However, the role of public policy is underexplored, and more ex-ante circularity assessments for RCSs, such as the one currently under consideration for automotive steel in the EU, are needed. Crucially, current RCSs typically target individual sectors and thus may overlook impacts across sectors. Dynamic material flow analyses (MFA) can be used to assess circularity potentials, with secondary material supply depending on the accumulation and release of in-use stocks.^24^ Recent MFA studies on steel show that product-relevant characteristics and quality constraints should be considered to avoid overestimating circularity potentials in mass-based models.^25,26^

Here, we assess the cross-sectoral circularity implications of an RCS for automotive steel in the EU, considering the principles of stock-flow modeling, scrap quality constraints, and the cross-sectoral dynamics outlined above. We develop the Policy Assessment of Recycled Content Standards (PARCS) model using dynamic MFA, comprehensively assessing uncertainties with multiple scenarios, a Monte Carlo simulation, and sensitivity analyses. We apply PARCS to the automotive (passenger cars) and building (residential and nonresidential buildings) sectors in the EU to simulate the steel stocks and flows. We choose the building sector because ELV steel scrap currently largely does not meet automotive steel requirements and is therefore redirected to it. The model assesses the circularity potential in each year over a near-term, policy-relevant timeline (2025–2050) with the new *Closed-Loop Post-Consumer Circularity Potential* (CLOP) indicator.

CLOP determines how much closed-loop post-consumer scrap arises in the EU and can potentially be used to produce new vehicles and buildings in the same year. CLOP is tailored to regulatory developments requiring a share of recycled content from closed-loop post-consumer sources in new products. By implementing a sector-coupled MFA approach, we specifically consider scrap quality constraints and assess how improving circularity in one area, the automotive sector, may impact circularity elsewhere, namely the building sector, thereby elucidating potential trade-offs between closed-loop recycling – where materials are recycled back into the same product system – and open-loop recycling – where recycled materials are used in a different product system.^27^ We quantify the cross-sector impact using CLOP^PLUS^, which, in addition to closed-loop post-consumer scrap, includes the scrap a sector receives from another sector.

Our contribution is 3-fold: first, our model is a novel sector-coupled approach to quantitative ex-ante CE policy assessments, illustrated with an RCS for closed-loop automotive steel, particularly considering scrap quality constraints and system-wide effects. Second, we help identify crucial processes and levers for policy intervention throughout the product life cycle to close material loops. Third, we provide a quantitative tool for estimating appropriate RCS levels over time and the parameters that influence them, including crucial uncertainties. These contributions could be valuable for researchers seeking to model and quantify the systemic effects of circularity interventions; for policymakers to identify levers for policy intervention within value chains and across sectors; and for industry practitioners who wish to prepare for potential RCSs or become CE frontrunners.

## 2 Methods

### 2.1 Model Overview

Dynamic MFA enables the analysis of material stocks and flows over time.^28^ Here, we develop the PARCS model, which simulates the steel demand and potential closed-loop post-consumer recycled steel supply in the automotive sector (1990–2060) and the building sector (1900–2100) in the European Union (EU27) using dynamic MFA. The goal is to determine the extent to which steel demand in each year and sector can be met with recycled steel from closed-loop post-consumer sources over a policy-relevant timeline (2025–2050). We do not include steel imports and exports; we consider only collection losses from used-car extra-EU net exports and ELVs with unknown whereabouts, the latter of which include extra-EU exports not tracked in official databases. We justify this simplification: a potential RCS may target cars sold in the EU, rather than those produced there. Hence, our research is interested in how much steel scrap from European ELVs, that is, from closed-loop post-consumer sources, could potentially cover steel demand in new cars registered in the EU.

The model simulates flows and stocks in two sectors (automotive and buildings), assuming three CE development scenarios (*BaseCE*, *HighCE*, and *LowCE*) and two cases (*no closed-loop intervention* and *automotive closed-loop intervention*) – including uncertainty bands from a Monte Carlo simulation. The two modeled sectors include, for each product, four processes (product production, product use phase, scrap allocation, and steel production) and multiple losses described below (fabrication, collection, recycling, and remelting). Both sectors are connected through a flow reflecting our sector-coupled approach: scrap from ELVs that cannot be used for automotive steel flows to the building sector. Finally, we calculate the closed-loop circularity potential using the new *Closed-Loop Post-Consumer Circularity Potential* (CLOP) and CLOP^PLUS^ indicators as defined below.

### 2.2 Modeling the Automotive and Building Sectors

Following dynamic MFA principles,^29^ Figure 1a shows the modeled processes, stocks, flows, and parameters in the automotive sector in the EU. In vehicle production (process 1), we use inflow-driven (historical) and stock-driven (future) modeling to calculate the number of vehicles and associated steel inflows based on historical vehicle registrations, projected vehicle stock, and historical and projected steel intensities per car. The vehicle use phase (process 2) estimates the ELVs and associated steel outflows entering the formal waste management system, based on a Weibull lifetime distribution that approximates vehicle survival rates,^30^ and collection losses, including extra-EU net exports of used cars and unknown whereabouts of ELVs. An analysis by Baron et al.^31^ for the EU helps grasp the scale of these losses: for 2019, they estimated used-car extra-EU imports at 340,000 vehicles, while used-car extra-EU exports at 970,000 vehicles.^31^ ELVs with unknown whereabouts, which reflect unreported extra-EU ELV exports as well as illegal treatments, amounted to 3,400,000 vehicles.^31^ In comparison, new registrations amounted to 14,810,000 vehicles. Details are provided in the Supporting Information.

**Figure 1:**
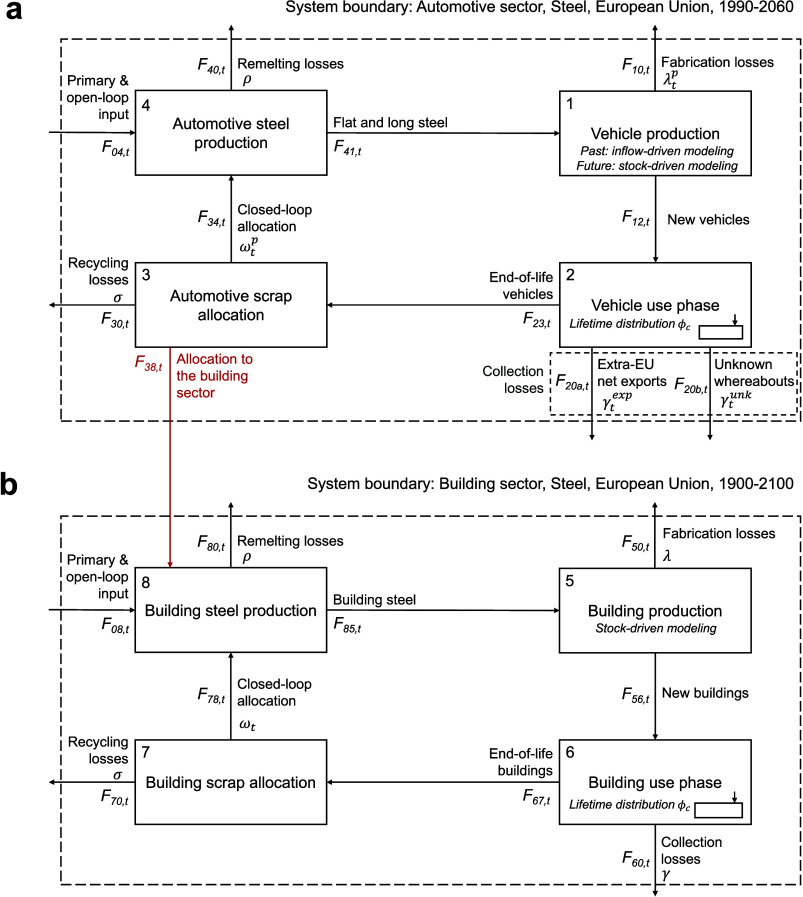
Graphical representation of the (a) automotive sector and (b) building sector in the EU modeled in PARCS. Index t for year, c for age-cohort, and p for products (flat or long steel) in the automotive sector. The building sector does not differentiate between flat and long steel.

Automotive scrap allocation (process 3) considers recycling losses that arise when steel scrap undergoes dismantling, shredding, and postshredding. The resulting available scrap is assigned to different destinations. We allocate scrap using a hierarchy that prioritizes value preservation and applications requiring higher-quality scrap: first, closed-loop flat steel; second, closed-loop long steel; and third, open-loop steel in buildings. The first two applications are subject to a maximum scrap use limit due to quality constraints. For the third, namely, buildings, we assume no limit. Finally, automotive steel production (process 4) calculates scrap in new vehicles after deducting remelting losses, arising from melting scrap to produce new steel products, and fabrication losses, when processing steel products into cars.

Figure 1b shows the modeled building sector. In building production (process 5), we use stock-driven modeling to calculate building and associated steel inflows based on the age-cohort distribution of the building stock in 2020 in the EU, the projected building stock, and the historical and projected steel intensity of buildings, differentiated between residential and nonresidential buildings. The building use phase (process 6) assumes a Weibull lifetime distribution and collection losses to determine the end-of-life buildings and associated steel outflows. Building scrap allocation (process 7) deducts recycling losses and assigns the available scrap to closed-loop steel production. We assume recycling losses that reflect a technical limit on how much scrap can be used for closed-loop building steel production after recycling. Eventually, steel production (process 8) determines the scrap, which can stem from end-of-life buildings or ELVs, in new buildings after remelting and fabrication losses.

### 2.3 Six Scenarios in the Automotive and Building Sectors

We model three developments of CE ambition: BaseCE, HighCE, and LowCE. BaseCE assumes an evolution based on historical patterns, current practices, and already-implemented policies. HighCE assumes more pronounced CE efforts than BaseCE, and LowCE assumes less-pronounced CE efforts than BaseCE. The developments represent possible futures for the world and affect our projections of the input parameters given in Table 1. One future is not necessarily more likely than the others; they help us understand how different data assumptions affect the model results.

**Table 1:** Overview of the Model’s Input Data and How the CE Development Scenarios HighCE and LowCE Compare to BaseCE^a^

				Comparison to BaseCE	
**Sector**	**Process**	**Input data**	**Unit**	**HighCE**	**LowCE**
Automotive	Vehicle production	Vehicle registrations^33^	Vehicles	*Historic only*	
		Vehicle saturation^17,34^	Vehicles/1000 inh.	↓	↑
		Population^35,36^	Inh.	=	=
		BEV share^33,37^	%	↑	↓
		Steel intensity BEV^38,39^	kg/veh.	↓	↑
		Steel intensity ICEV^38,39^	kg/veh.	↓	↑
		Share flat vs long BEV^38^	%	=	=
		Share flat vs long ICEV^38^	%	=	=
	Vehicle use phase	Lifetime^30^	Years	↑	↓
		Weibull shape parameter^30^	-	=	=
		Collection losses: Unknown whereabouts^31,40^	%	↓	↑
		Collection losses: Extra-EU net exports^31,40^	%	=	=
	Scrap allocation	Recycling efficiency^31,41^	%	=	=
		Max. closed-loop flat^32,38^	%	*NCI: 0%*	
				*ACI: from 0% in 2025 to 60% in 2050*	
		Max. closed-loop long^38^	%	Faster ↑	↓
	Steel production	Remelting losses^13,42,43^	%	=	=
		Fabrication losses flat^44^	%	Faster ↓	↑
		Fabrication losses long	%	=	=
Buildings	Building production	Res. building stock in 2020 ^45^	m^2^	*Historic only*	
		Nonres. building stock in 2020 ^45^	m^2^	*Historic only*	
		Projected res. buildings^46^	m^2^/inh.	↓	↑
		Projected nonres. buildings^46^	m^2^/inh.	↓	↑
		Steel intensity res. buildings^46,47^	kg/m^2^	↓	↑
		Steel intensity nonres. buildings^46,47^	kg/m^2^	↓	↑
		Population^35,36^	Inh.	=	=
	Use phase	Lifetime^48^	Years	↑	=
		Weibull shape parameter^48^	-	=	=
		Collection losses^13^	%	=	=
	Scrap allocation	Recycling efficiency^13^	%	↑	=
	Steel production	Remelting losses^13,42,43^	%	=	=
		Fabrication losses^13,49^	%	=	=

a ↓ means lower than, ↑ means higher than, and = means equal to BaseCE. Assuming a no closed-loop intervention (NCI) case, the maximum share of closed-loop postconsumer scrap in new automotive flat steel is 0%. Assuming the automotive closed-loop intervention (ACI) case, the share increases from 0% in 2025 to 60% in 2050. The NCI and ACI cases are modeled for all scenarios.

Our model is agnostic toward the three futures’ drivers. Parameters may evolve without policy intervention; however, the model provides policymakers with a perspective on how parameters affect outcomes and potential policy levers. For HighCE, these include speeding up technology transitions (e.g., from internal combustion engine to battery electric vehicles), increasing material efficiency by achieving the same service provision with fewer products (e.g., limiting the increase in vehicle ownership) and less material input (e.g., lightweighting vehicles), extending product lifetimes (e.g., building longer lasting cars), reducing collection losses (e.g., implementing traceability initiatives to tackle illegal dismantling of cars), and improving manufacturing efficiency (e.g., reducing fabrication losses when processing sheet metal in cars).

Closed-loop circularity in the automotive sector is currently limited by the inability to use most post-consumer scrap for high-quality flat steel due to contamination by tramp elements, particularly copper.^11^ However, recent trials have indicated that improved ELV dismantling, sorting, and postshredder technologies can substantially reduce copper content and improve scrap quality.^32^ Given the significance of this potential development, we model two main cases across all three CE scenarios. In the *no closed-loop intervention* (NCI) case, we assume that closed-loop post-consumer scrap cannot be used to produce automotive flat steel, as is currently the case. In the *automotive closed-loop intervention* (ACI) case, we model the intervention of improving scrap quality, as described below. In the Results section, we present the combination of three CE developments and two cases, yielding six distinct scenarios per sector.

### 2.4 Modeling an Automotive Closed-Loop Circularity Intervention

The automotive scrap allocation process is at the center of the model. Currently, virtually no post-consumer scrap from ELVs can be used to produce flat steel of sufficient quality for new vehicles.^38^ Flat steel makes up the largest share of steel in vehicles. Hence, increasing the maximum post-consumer scrap use rate for flat steel is crucial to improving closed-loop circularity in the automotive sector. Increasing the rate requires improving the scrap quality. Given the high sensitivity of this assumption, we do not model an improvement within just one of the three CE scenarios. Instead, efforts to increase scrap quality can happen in all three scenarios, and we define the separate ACI case.

Globally, tramp elements, especially copper, increasingly contaminate steel scrap.^50^ If the copper concentration is too high, scrap cannot be used for high-quality applications such as flat steel in the automotive sector. Due to losses at every recycling loop, most scrap today ends up in buildings, which have lower quality requirements.^13^ Daehn et al.^11^ highlighted the risk of a growing stock of unusable scrap. Part-based sorting and alloy management can reduce the quality loss, as shown in a study of ELVs in Japan.^51^ However, achieving automotive closed-loop circularity may require multiple interventions along the supply chain, such as improved disassembly, shredding, sorting, and chemical extraction, increased copper tolerance, and reduced copper content.^11^

A recent trial by Gross and Hermine^32^ in France has shown that improved dismantling techniques before shredding, namely manual wiring removal, can reduce the copper content in steel scrap from ELVs to 0.09% - compared to the current average of 0.4%. This concentration is below the 0.15% threshold required for automotive flat steel production.^32^ Currently, it is possible to use up to 60% high-quality post-consumer scrap for flat steel production via the Electric Arc Furnace (EAF) in the United States.^32^ We assume this holds for the EU as well and that the maximum post-consumer scrap use rate can be increased to 60% for closed-loop automotive flat steel production. Initially, the Blast Furnace – Basic Oxygen Furnace (BF-BOF) could accept higher-quality scrap. The BF-BOF route is predominant in the EU and can accept up to 20% high-quality scrap as coolant, which is currently covered by prompt scrap. In the future, reaching a maximum scrap use rate of 60% would require the deployment of upgraded EAF routes to accept more scrap^52^ and sufficient availability of Direct Reduced Iron (DRI).^32^ We assume this improvement in the ACI case will be gradual, so we model a logistic improvement (S-curve) from 0% in 2025 to 60% in 2050, as 2050 represents a critical year for sustainability-related policymaking. In the Supporting Information, we discuss the sensitivity of this case by showing five additional variations of the ACI case.

Notably, the model does not explicitly track quality changes in scrap diverted to the building sector under the ACI case, assuming implicitly that its quality remains unchanged. Depending on the intervention, quality could in fact improve (if copper is removed at source through dismantling before shredding) or worsen (if postshredder sorting selectively diverts cleaner fractions to the automotive sector), leaving a lower-quality residual for buildings and implying higher recycling losses than modeled. This simplification is described in more detail in the Supporting Information, and its implications are discussed in section 4.1.

### 2.5 Analyzing the Results and Assessing the Closed-Loop Circularity Potential

Circularity can be calculated in many ways, using “established” methods such as the Recycled Content (RC) indicator^53^ and the Material Circularity Indicator (MCI),^54^ which includes aspects such as product longevity. The Circularity Potential (c^rec^)^55^ and the closed-loop circularity rate (CLCR)^56^ – in simplified terms – are a “closed-loop” version of RC, expressing how much recycled materials can contribute to material demand from the same sector and product group. An overview of additional indicators can be found in the supplementary materials of a paper by Reichert et al.^57^ The diversity of indicators highlights a lack of consistency in academic research. Reichert et al.^57^ proposed the Product-oriented Closed-loop Material Indicator (PCMI), which addresses regulatory developments requiring the declaration of closed-loop recycled content in new products, such as the EU ELV Regulation Proposal.^7^ The PCMI, however, is mainly suitable for compliance and highly accurate declarations of closed-loop recycled content, requiring detailed data from all actors in a product’s value chain.

Here, we propose an approach comparable to the CLCR – but for a final product before it enters the use phase. The Closed-Loop Post-Consumer Circularity Potential (CLOP) first introduced here describes how much closed-loop post-consumer waste could be incorporated into a new product, aligned with the RCS proposed by the European Commission,^7^ which sets targets for the amount of recycled steel from ELVs in new cars. The CLOP relates the possible provision of steel from closed-loop post-consumer sources in a year to the total steel demand for new products in that year. This means that all processes P1–8 in Figure 1 happen in the same year.

Generally, we define *CLOP* for sector *S* at year *t*, with steel demand for new products *D* and potential supply of recycled content from closed-loop post-consumer sources *R*, as follows:

$$CLOP_{t}^{S}=\frac{R_{t}^{S}}{D_{t}^{S}}$$

For the automotive sector *A*, *CLOP* at year *t*, for product *p* (flat steel *FS* and long steel *LS*), with recycling losses σ, closed-loop allocation factor ω_*t*_^*p*^, remelting losses ρ, and fabrication losses λ_*t*_^*p*^, is calculated as follows in our model:

$$\begin{array}{c} CLOP_{t}^{A}=CLOP_{t}^{FS}+CLOP_{t}^{LS}\\ with\;CLOP_{t}^{p}=\frac{F_{23,t}\left(1-\sigma \right)\omega_{t}^{p}\left(1-\rho \right)\left(1-\lambda_{t}^{p}\right)}{F_{12,t}}\;,and\;p\in \left\{FS,LS\right\} \end{array}$$

In the building sector *B*, we do not differentiate between flat steel and long steel. Hence, *CLOP* at year *t*, with remelting losses ρ, and fabrication losses λ, is calculated as follows:

$$CLOP_{t}^{B}=\frac{F_{78,t}\left(1-\rho \right)\left(1-\lambda \right)}{F_{56,t}}$$

As a novelty in this study, we attempt to understand sector interactions. Hence, for the building sector, we quantify the cross-sectoral *CLOP*_*t*_^*PLUS*^ with flow *F*_38_ representing the scrap diversion from the automotive industry to the building sector due to quality constraints:

$$CLOP_{t}^{PLUS,B}=\frac{\left(F_{38,t}+F_{78,t}\right)\left(1-\rho \right)\left(1-\lambda \right)}{F_{56,t}}$$

Strictly speaking, *CLOP*_*t*_^*PLUS*,*B*^ is no longer a closed-loop indicator; rather, it explicitly includes flows from the automotive sector.

### 2.6 Quantifying Uncertainties with a Monte Carlo Simulation

We quantify parameter uncertainty with a Monte Carlo (MC) simulation – a common approach for MFA.^58^ We run the model 10,000 times. In each run, we draw random samples from predefined probability distributions (normal and log-normal) for the relevant input parameters, resulting in a distribution of possible model outcomes. We choose the standard deviations for each parameter according to the following criteria. First, if we have multiple competing sources for a parameter, we calibrate the standard deviation by interpreting the envelope spanned by all sources as a 95% uncertainty interval. In other cases, decision criteria include, among others, (i) relatively lower standard deviation for reliable data sources such as Eurostat and academic research, (ii) relatively higher standard deviation in the far future if scenario uncertainty is not covered, (iii) relatively higher standard deviation for speculative values, for instance, those based on industry reports. Additional details about the MC simulation are available in the Supporting Information. The chosen approaches and standard deviations for all input variables are in the PARCS repository. Our MC simulation gives 10,000 results. We report the medians and, where appropriate, the 95% uncertainty intervals (2.5th–97.5th percentiles), i.e., 95% of simulation outcomes are within that range.

### 2.7 Model Limitations

A critical methodological choice is that we do not consider prompt or new scrap, which can be pre-consumer waste or reutilization flows. A graphical representation of the different scrap types is available in the Supporting Information. “Pre-consumer waste” arises before the product use phase, i.e., in production and manufacturing processes. It counts as recycled content under ISO.^59^ “Reutilization flows” are not considered “waste” and therefore do not count as recycled content.^59^ Like pre-consumer waste, their quality is high and can be regarded as a manufacturing inefficiency.^60^ Therefore, value chain actors are typically incentivized to reduce prompt scrap to whatever extent possible. Furthermore, prompt scrap is reused and recycled extensively thanks to its relatively high quality. Thus, keeping prompt scrap in the loop does not require additional policy intervention – unlike old scrap or post-consumer waste, which is typically of lower quality due to mixed scrap fractions in recycling and waste management processes. This selection aligns with regulatory developments, including the European Commission’s ELV Regulation Proposal, as most RCSs target post-consumer waste.^6^ For these reasons, our research focuses on the potential of closed-loop post-consumer scrap to cover steel demand. Future work, however, could address how different system boundaries affect the results.

Additional opportunities for improvement, leading to new research avenues, include the impacts of considering steel imports and exports on the model results as well as related ramifications, such as geopolitics and material flow resilience. Furthermore, our model currently assumes that scrap is prioritized for high-quality applications. Alternative hierarchy logics could be tested or extended to optimization problems. Moreover, the model does not explicitly track quality changes in scrap diverted to the building sector under the ACI case, which may constitute an upper bound on building-sector circularity depending on the intervention, as discussed in sections 2.4 and 4.1.

A final point on validating PARCS: a direct output-level validation is constrained for two reasons. First, CLOP is a potential indicator that quantifies the maximum closed-loop post-consumer recycled content achievable under idealized allocation assumptions and not current practice. We could not find EU-level data on the actual closed-loop recycled steel content in new vehicles for a formal comparison. Second, the model tracks steel demand based on new car registrations rather than production, making a direct comparison to sector-level statistics less meaningful. Notwithstanding these constraints, three consistency checks support the model calibration. The modeled EU passenger car stock of approximately 270 million vehicles in 2024 is consistent with the Eurostat-reported stock of approximately 260 million in 2024,^61^ validating the stock-flow approach. The modeled gross steel demand of approximately 15 Mt in new registered passenger cars in 2022 compares with an automotive steel demand of 23.7 Mt reported by Hill et al. (2024),^39^ with the difference reflecting that their figure captures steel consumed in EU vehicle manufacturing, potentially including exported vehicles as well as commercial vehicles and other nonpassenger-car applications, rather than steel embodied in passenger cars registered in the EU as modeled here. Finally, the CLOP in the NCI case of approximately 6% in 2025 is of the same order of magnitude as the current post-consumer scrap use rates (both open- and closed-loop) of 11.3% for ICEVs and 5.2% for BEVs reported by Sutter et al. (2025),^38^ noting that CLOP reflects the theoretical potential under idealized closed-loop allocation assumptions rather than observed practice.

## 3 Results

The results show that improving the scrap quality from ELVs is the decisive lever for automotive closed-loop circularity. This improvement comes at the cost of a reduced scrap availability for the building sector. The following subsections build toward this finding: section 3.1 describes how scrap quality constraints and CE development shape steel flows in the automotive sector; section 3.2 shows how the building sector is affected by both its own dynamics and scrap diversion from the automotive sector; and section 3.3 quantifies the circularity implications of these dynamics across both sectors using the CLOP and CLOP^PLUS^ indicators.

### 3.1 Steel Flows and Scrap Quality Constraints in the Automotive Sector

Figure 2a shows the median steel flows in the automotive sector for the three scenarios in the NCI case. In BaseCE, steel in ELVs suffers significant losses before becoming usable scrap. Losses include, in descending order, collection losses due to ELVs with unknown whereabouts that do not enter the formal waste management system, collection losses due to extra-EU exports, and recycling losses. The recycling losses are negligible in terms of mass percentage. Still, crucially, the resulting scrap cannot be used to produce automotive flat steel, which accounts for the largest share of steel in cars, because its quality is insufficient. A small share of scrap can be used for automotive long steel, subject to remelting and fabrication losses. Most scrap is diverted to the building sector, where the quality constraints are laxer. Thus, the remaining supply of recycled steel from ELVs pales compared with the demand for steel in new vehicles.

**Figure 2:**
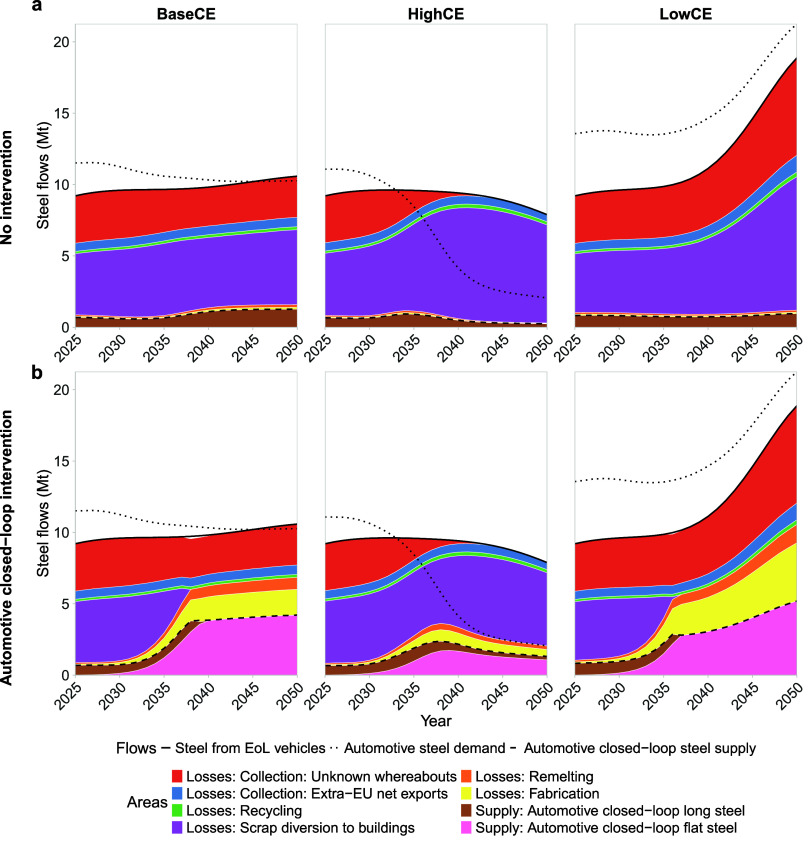
Steel flows in the automotive sector per year in three CE development scenarios (BaseCE, HighCE, and LowCE) and two cases (a) No closed-loop intervention and (b) Automotive closed-loop intervention. Deducting different types of losses from the “steel from End-of-Life (EoL) vehicles” leads to the “automotive closed-loop steel supply”, which includes closed-loop flat steel and long steel production. The values are the median from the Monte Carlo simulation. Uncertainty bands are provided in the source data.

In HighCE, the demand for automotive steel decreases over time. We model strong CE measures with improvements in material efficiency, including fewer cars to fulfill the same mobility demand and vehicle lightweighting, and a more rapid proliferation of BEVs, which contain less steel than ICEVs. Furthermore, we assume that cars are built to last for longer. Counterintuitively, in the HighCE scenario, the absolute scrap diversion to buildings is higher over time than that in BaseCE. The reason for this is our assumption that collection losses due to unknown whereabouts will be addressed, for instance, through improved tracking and collection systems, thereby increasing scrap availability. This scrap can only be used in the building sector because of quality constraints for automotive flat steel production. In LowCE, automotive steel demand increases over time, driven by the assumption that the number of new vehicles and their steel content will rise, following a trend of more and heavier cars. At the same time, we model that vehicles will be used for shorter periods, thereby increasing car demand in the long term.

Figure 2b shows the ACI case. This case assumes that copper wires are manually dismantled from ELVs before entering the recycling process, and ELVs are treated separately from other scrap. The result is scrap of higher quality, which we assume is allocated to automotive flat steel production as a priority. In BaseCE, after 2035, this new allocation comes at the expense of scrap diversion to building steel production and automotive long steel production. However, fabrication losses for automotive flat steel are high before steel ends up in new vehicles. In HighCE, steel demand decreases to an extent that all of the available scrap can be used, up to a technical limit, for automotive flat steel and long steel production. Hence, counterintuitively, scrap diversion from the automotive to the building sector persists despite higher CE efforts. LowCE behaves similarly to BaseCE, albeit with higher steel demand, for the same reasons as those in the NCI case in Figure 2a.

These results show that regardless of CE development scenarios the quality constraints on automotive flat steel production remain a binding factor: most ELV scrap is diverted to the building sector, with only the ACI case substantially redirecting scrap flows toward automotive steel production.

### 3.2 Steel Flows and Automotive Scrap Diversion in the Building Sector

Figure 3a shows the median steel flows in the building sector for three scenarios in the NCI case with three crucial insights. First, the demand for steel for buildings is typically higher than that for cars. Second, the supply of closed-loop recycled steel from end-of-life buildings is similar in all three scenarios. The building sector shows signs of inertia, as building lifetimes are long and the room to improve collection, recycling, remelting, and fabrication losses is limited. Third, the supply of recycled steel from end-of-life buildings and vehicles exceeds the demand for new building steel in HighCE. Figure 3b displays the ACI case. Here, the flows are similar to those in the NCI case, except for varying amounts of scrap from the automotive sector. Most interestingly, scrap diversion to buildings persists only in HighCE over time, as decreasing automotive steel demand generates surplus scrap that cannot be absorbed within that sector.

**Figure 3:**
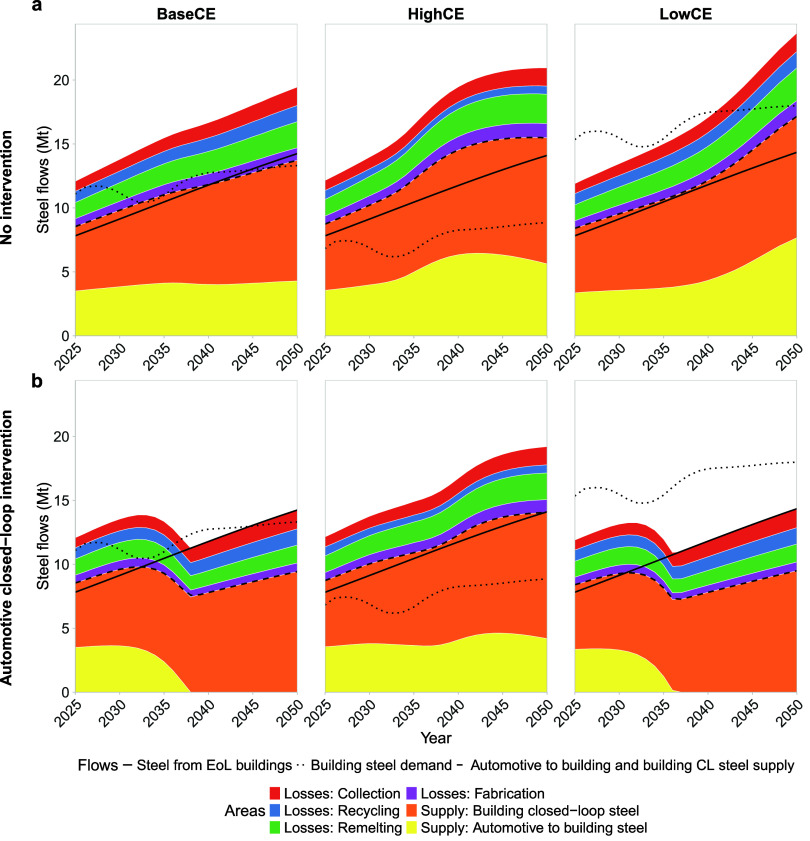
Steel flows in the building sector per year in three CE development scenarios (BaseCE, HighCE, and LowCE) and two cases (a) No closed-loop intervention and (b) Automotive closed-loop intervention. Deducting different types of losses from the “steel from End-of-Life (EoL) buildings” and from the scrap diverted from the automotive sector (Figure 2) leads to the “automotive-to-building and building closed-loop (CL) steel supply”. The values are the median from the Monte Carlo simulation. Uncertainty bands are provided in the source data.

### 3.3 Cross-Sectoral “Circularity Battle” in the Automotive and Building Sectors

We assess circularity in the automotive and building sectors using the new Closed-Loop Post-Consumer Circularity Potential (CLOP) and CLOP^PLUS^ indicators, including MC uncertainty bands, as shown in Figure 4.

**Figure 4:**
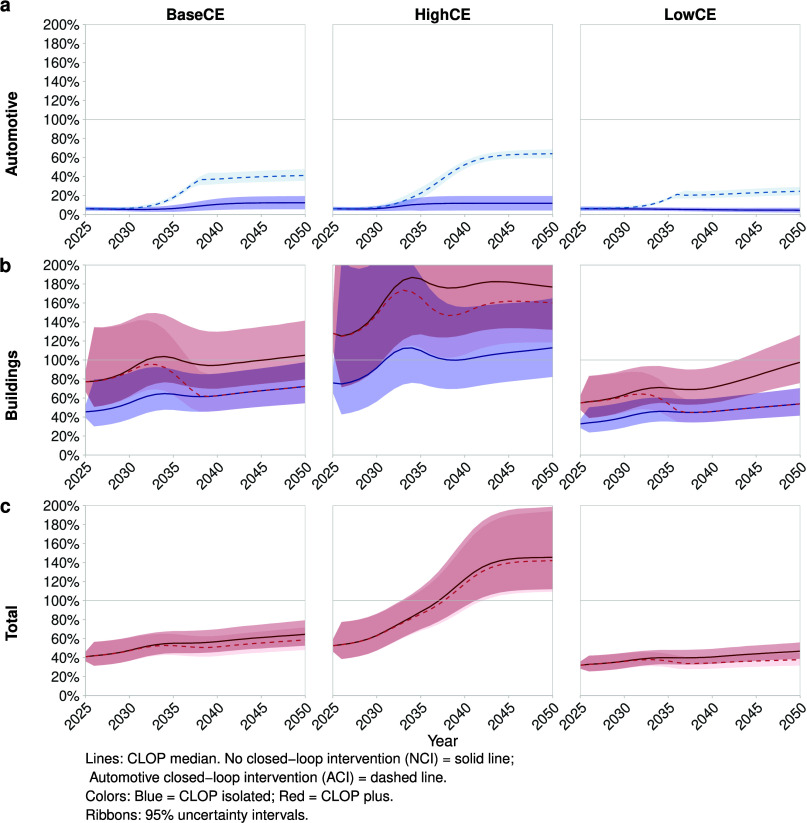
Closed-Loop Post-Consumer Circularity Potential (CLOP) for three CE development scenarios (BaseCE, HighCE, and LowCE) and three perspectives (a) Automotive, (b) Buildings, and (c) Total, where “Total” is the combination of the automotive and building sectors. “Plus” in the building industry includes the scrap diversion from the automotive industry. A value higher than 100% means that more scrap would be available from post-consumer sources than is necessary to cover steel demand in the EU. The Supporting Information provides the source data, including the median values and 95% uncertainty intervals (2.5th–97.5th percentiles) for all years.

The values for 2035 and 2050, including the deltas between the NCI and ACI cases, are shown in Figure 5. The results show that circularity is typically highest in HighCE, intermediate in BaseCE, and lowest in LowCE. Assuming NCI, the circularity potential is similar for the automotive sector in all three scenarios. Without a dedicated effort to improve scrap quality, circularity is poised to stay low, independent of CE developments, ranging between 4% and 12% (2–19%) in 2035–2050. Only with an ACI could circularity increase drastically. In 2035, closed-loop circularity could be 18% (15–20%) in BaseCE, 22% (17–28%) in HighCE, and 17% (15–18%) in LowCE. In 2050, differences between scenarios will be more pronounced, with circularity increasing to 41% (35–48%) in BaseCE, 64% (59–69%) in HighCE, and 24% (20–29%) in LowCE. These results show that only in HighCE does the improvement in scrap quality and the associated higher use for automotive flat steel production (up to 60%) almost fully translate into the highest possible CLOP rates. In BaseCE and LowCE, scrap availability is a limiting factor instead. A key insight is that improving the quality of ELV scrap is a precondition for higher closed-loop circularity rates in the automotive sector – but the improvement is most evident when it occurs in parallel with complementary circular economy measures.

**Figure 5:**
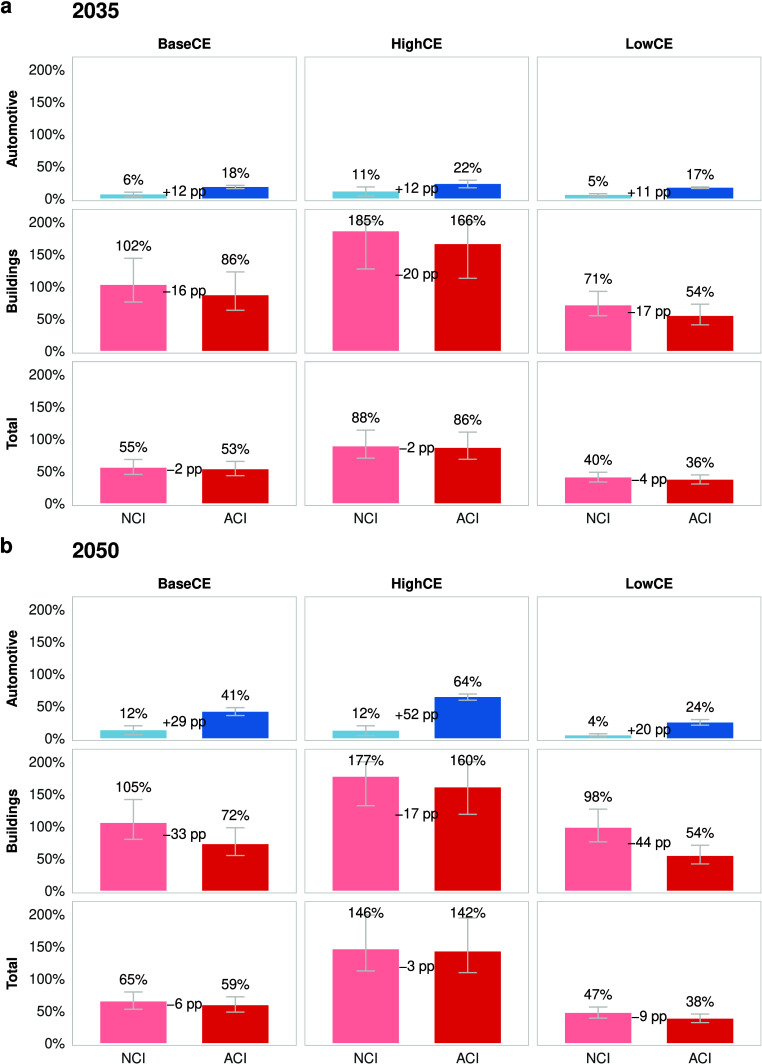
Closed-Loop Post-Consumer Circularity Potential (CLOP) in % in the automotive, buildings, and total sectors in (a) 2035 and (b) 2050. Blue represents CLOP for an isolated sector, red represents CLOP^PLUS^ – i.e., the building sector includes the scrap diversion from the automotive industry. NCI is the no closed-loop intervention case, and ACI is the automotive closed-loop intervention case. Values above bars are medians with 95% uncertainty intervals as error bars (2.5th–97.5th percentiles). Values between bars are the delta between NCI and ACI in percentage points (pp).

For buildings, we observe that the scrap diversion from the automotive sector enables higher circularity rates than in an isolated case. Assuming the automotive sector improves its own closed-loop circularity, the building sector’s open-loop circularity decreases by up to 44 percentage points and equals the isolated case by 2035–2040 in BaseCE and LowCE. In the HighCE scenario, however, different patterns emerge. On the one hand, in 2050, the automotive sector improves its own circularity the most, while reducing the building sector’s circularity the least. On the other hand, closed-loop circularity in the building sector will exceed 100% in the isolated case by 2031. Open-loop circularity may already be above 100% in 2025, accounting for scrap diversion from the automotive industry. A value higher than 100% means that more scrap would be available from post-consumer sources than is necessary to cover steel demand in the EU.

Further sensitivity analyses of the ACI case in Figure 13 of the Supporting Information, with varying speeds and magnitudes of scrap quality improvement, show how the direction of the trade-off is robust across all variants: automotive CLOP increases and building CLOP^PLUS^ decreases under all ACI specifications, with the magnitude depending on the speed and ceiling of scrap quality improvement and on scrap availability.

A combined “total” view of the automotive and building sectors shows that overall circularity decreases slightly as the automotive sector intensifies its circularity efforts. The decrease is due to CLOP being measured at the new product level, which aligns with regulatory requirements. Scrap used in the automotive sector undergoes higher fabrication losses than scrap used in the building sector. For the combined total view, an alternative CLOP indicator at the “gross” level, i.e., defined before remelting and fabrication losses, shows that the values remain unchanged in both cases (see the Supporting Information).

Overall, these results reveal a cross-sectoral trade-off: improving closed-loop circularity in the automotive sector reduces scrap availability and the building sector’s open-loop circularity, with the magnitude depending on the CE development scenario and the automotive sector’s scrap quality improvement.

## 4 Discussion

### 4.1 How to Increase Circularity in the Automotive Sector and Its Impacts on the Building Sector

Without improving the quality of recovered scrap from ELVs, closed-loop circularity in the automotive sector is poised to remain low – regardless of the adoption of other CE measures, such as improved material efficiency and collection efforts. When enhancing the quality of recovered scrap, however, circularity increases the most under a high adoption level of other CE measures. Ultimately, improving scrap quality and using it for automotive flat steel depends on multiple actors: carmakers can reduce copper use in new vehicles and design them in a way that simplifies disassembly and dismantling when they turn into ELVs; dismantlers can manually remove more copper; shredder and postshredder operators can use new sorting and separation techniques; and steel producers can improve their technology to increase copper tolerance. These measures can be costly and will take time to materialize. Information sharing between actors, such as digital product passports and traceability initiatives, could be helpful.^62^ Policymakers must assess which levers are most effective for improving scrap quality.

A sector that improves its closed-loop circularity may merely keep more scrap for itself that would otherwise be diverted to another sector. Most interestingly, in our analysis of steel, this “redistribution” pattern is less pronounced in a scenario of high CE adoption. The higher the CE adoption in the automotive sector, the more likely scrap diversion will persist, as more surplus scrap becomes available due to lower automotive steel demand. Crucially, the quality implications of this redistribution pattern may depend on the intervention. If scrap quality is improved through deeper dismantling before shredding – as assumed in this model – copper is removed at the source, benefiting both sectors. If scrap quality is improved through postshredding technology, cleaner fractions may be diverted to the automotive sector, leaving the residual available for buildings of lower quality. Future research should explicitly track scrap quality characteristics across the recycling chain to disentangle the effects of different quality interventions.

Another way of framing the redistribution pattern, however, is that improving circularity in the automotive sector does not necessarily take anything away from the building sector; instead, it empowers the automotive industry to take back what is its own. Overall, we argue that all sectors must contribute to preserving the value of materials over multiple life cycles, as we find a high risk of saturation with low-quality scrap in the building sector, potentially leading to unusable scrap.

### 4.2 Designing Circular Economy Policies and Setting Recycled Content Standards

Many levers exist to increase recycled content from closed-loop post-consumer waste throughout a product’s life cycle. Thus, a policy mix may be needed, rather than just a single instrument. However, designing CE policy mixes is complex. On the one hand, there is a need to coordinate *within* sectors. Multiple actors and processes should be incentivized, especially to improve scrap quality and increase collection efforts, material efficiency, and sufficiency. On the other hand, coordination *across* sectors is needed. What is best for one industry may not be best for another one. Steering recycled content at the product level within individual sectors, as shown by our CLOP indicator, may not be sufficient to promote system-wide circularity. Alternatives could be assessing circularity using the CLOP at the gross input level, similar to the recycled content indicator,^53^ or minimizing surplus scrap and primary material in the system.

For policymakers interested in setting RCSs, choosing an appropriate RCS level specifically from closed-loop post-consumer sources is challenging, as it depends on how CE adoption evolves. Policymakers can affect the level of CE adoption through targeted measures, but this may also depend on macroeconomic and industry developments, which are highly uncertain, as indicated by our Monte Carlo simulation and sensitivity analyses. Therefore, a gradual introduction with stepwise increasing RCS levels over time and regular revisions may be helpful. Most importantly, RCSs should not come at the expense of other CE strategies, such as lifetime extension, reuse, and remanufacturing. Finally, RCSs should avoid other potential unintended consequences, for instance, when users targeted by a steel RCS replace steel with aluminum that an RCS does not target.

### 4.3 Industrial Ecology Tools Should Inform Circular Economy Policies

Using dynamic MFA, our study shows the importance of industrial ecology tools in quantifying the circularity impacts of CE policies. PARCS considers only material circularity. Higher circularity contributes to sustainability in many cases, and mass-based metrics are often used as accessible proxies by policymakers and companies. However, mass-based metrics should be interpreted alongside impact-based methods.^63^ Established tools, such as life cycle sustainability assessments and techno-economic analyses, must be used to estimate impacts across sustainability’s environmental, social, and economic dimensions. These tools should be combined for an integrated study of potentially competing effects, and researchers could assess the impacts of closed-loop recycling: for instance, under what circumstances is it worth putting in extra effort for recycling steel with higher quality and keeping it in a closed loop rather than accepting quality losses and diversion to other sectors?

Systemic analyses should receive more attention, given the CE’s intra- and intersectoral complexity. Examples of tools include system dynamics and consequential life cycle assessments. The latter could be useful for understanding how closed-loop recycling in one sector affects marginal production technologies: For instance, advancing closed-loop circularity in the automotive industry may incentivize the deployment of advanced scrap-based secondary production routes, thereby replacing primary production. Accepting the diversion of scrap from the automotive sector to the building sector may not displace the primary route to the same extent, but rather the secondary route, fueled with other scrap sources.

This paper shows how a sector-coupled approach to dynamic MFA can be used to assess circularity across sectors rather than in isolation. Thus, PARCS provides an important first step for designing CE policies that benefit the whole system.

## Supplementary Material





## Data Availability

This study analyzes existing publicly available data. Details about the input data are in the Supporting Information. This study’s input data and code are available on GitHub (https://github.com/redeamm/PARCS) and permanently archived on Zenodo (https://doi.org/10.5281/zenodo.21431137). Source data for Figures 2, 3, and 4 are provided with this paper.

## Notes

Details about the figure source data (XLSX)
Details about the method and input data (PDF)
