# Supplementary material for: Circularity Policy Interventions in One Sector Can Reduce Circularity in Others
Source: Environ Sci Technol. 2026 Jul 22;60(30):20727–39. doi: 10.1021/acs.est.6c05815 (PMC13449893; doi:10.1021/acs.est.6c05815)
Supplement: Supplementary file 1 [file es6c05815_si_002.pdf]

Supporting Information

Circularity policy interventions in one sector can reduce circularity in others

Mattia Maeder[1]*, Dominik Reichert[1], Magnus Fröhling[1]

[1] Technical University of Munich (TUM), TUM Campus Straubing for Biotechnology and Sustainability, Chair of Circular Economy and Sustainability Assessment, Am Essigberg 3, 94315 Straubing, Germany

* Corresponding author: mattia.maeder@tum.de

Contents

1 Dynamic Material Flow Analysis ... 1

2 Modeling the automotive sector ... 1

3 Modeling the building sector ... 10

4 Modeling an automotive closed-loop circularity intervention ... 15

5 Quantifying uncertainties with a Monte Carlo simulation ... 17

6 Definitions of scrap ... 17

7 Additional results ... 18

References ... 21

1 Dynamic Material Flow Analysis

The model is a dynamic MFA. In a nutshell, dynamic MFA enables the analysis of material flows and stocks over time. Typically, there are two logics: inflow-driven and stock-driven modeling[1].

With inflow-driven modeling, the inflow I in year t is known. I_t equals the initial stock S of an age-cohort c where $c = t$. For any age-cohort, stocks where $t < c$ do not exist. The future stocks of an age-cohort, i.e., when $t > c$, can be calculated using the survival rates derived from a Weibull lifetime distribution. In each year $t > c$, the outflows O_t of an age-cohort c can be calculated by subtracting S_t from S_{t-1}. In a year t, the total stock of all age-cohorts can be calculated with the previous year's total stock S_{t-1}, the inflow I_t, and the total outflows O_t, as follows:

$$S_t = S_{t-1} + I_t - O_t$$

The total stock S_{t-1} is the sum of stocks in year $t - 1$ of all age-cohorts $c \leq (t - 1)$. The total outflows O_t are the sum of outflows in year t of all age-cohorts $c < t$.

With stock-driven modeling, the total stock S in year t is known. The individual age-cohorts' stocks and outflows requires the survival rates derived from a Weibull lifetime distribution. Furthermore, the calculation must start with time step 0, or the distribution of age-cohorts in year t must be known. Subsequently, in each year t, the required inflow I_t results from the total stock S_t, the previous year's stock S_{t-1}, and the total outflows O_t as follows:

$$I_t = S_t - S_{t-1} + O_t$$

2 Modeling the automotive sector

Figure 1 shows the automotive sector, including all the processes, stocks, and flows. The following describes the assumptions and data inputs for processes 1, 2, 3, and 4. We highlight that there are thousands of actors in the value chain (steel producers, component suppliers, carmakers, dismantlers, recyclers, intermediate traders, exporters, and importers), making the calculation of the "actual" closed-loop recycled content in new products highly complex[2]. However, modeling such a system is challenging due to limited data and computational resources. Furthermore, we argue that reducing model complexity helps to better understand the most crucial patterns for policy analysis. Here, we provide a reasonable simplification, following a "what-if" approach to determine the potential automotive closed-loop circularity.

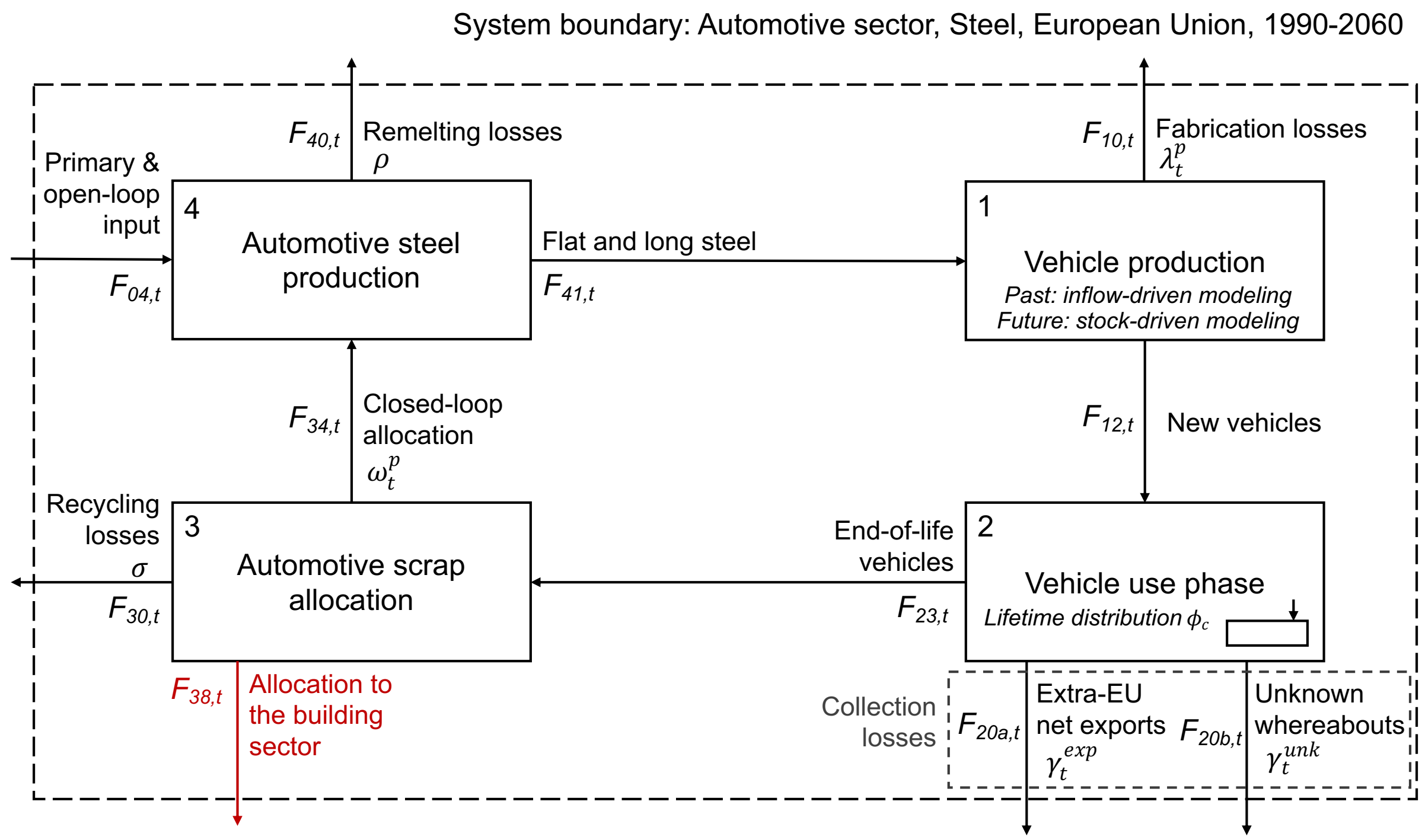

Figure 1: Automotive sector in PARCS.

2.1 Vehicle production

We calculate the car and associated steel inflows F_{12} into the vehicle use phase based on historical vehicle registrations, projected vehicle stock, and historical and projected steel intensity per car. The input data is in Figure 2.

For the historical simulation through 2023, we use country-level car registration statistics from Eurostat[3] and sum them for the EU27. Some countries have missing values in some years, which we extrapolate using forward filling when possible, or backward filling when no values are available from previous years. The car registrations enable inflow-driven MFA modeling.

We perform stock-driven modeling for the future simulation from 2024 until 2060, i.e., we must meet a projected car stock. We calculate the historic car ownership rate (cars per 1000 inhabitants) by dividing the endogenously[i] calculated historic car stock by the historic population from Eurostat[4]. Then, we assume a linear increase or decrease from 2024 to a saturation level in 2060. We set a saturation level of 600 in BaseCE, based on a logarithmic forecast of the 2013-2023 car ownership rates in the EU27 until 2050, according to Eurostat[5]. For HighCE, we assume a saturation level of 570, based on the current car ownership rate in the EU[5]. Therefore, it represents a stagnation in car demand, in which a decrease in countries with currently high car ownership rates offsets an increase in countries with currently low car ownership rates. The latter could be when mobility demand is met by fewer cars, for instance, thanks to the proliferation of car sharing and carpooling. For LowCE, we assume a saturation level of 700[6]. To calculate the projected car stock, we multiply the projected car ownership rate by the projected population from Eurostat[7].

[i] Endogenous means that values are calculated “internally” by the model. Exogenous means that values are deterministic and provided “externally” to the model.

The dataset contains values up to 2100 at 5-year intervals, so we perform spline interpolation for the years in between – allowing a smooth transition rather than sawtooth steps every 5 years.

We multiply the vehicles produced yearly by the steel intensity to calculate the steel inflows F_{12} into the use phase. To determine the steel intensity, we use the share of Battery Electric Vehicles (BEVs) versus Internal Combustion Engine Vehicles (ICEVs), the amount of steel in BEVs and ICEVs, and the share of flat steel versus long steel in BEVs and ICEVs.

For the historical BEV share, we use values from Eurostat[3,8]. For the future projection in BaseCE, we assume a logistic increase to 90% in 2035. We base this assumption on the current EU regulation requiring 100% "zero-emission" vehicles by 2035 [9], but consider a 10% ICEV share fueled with "zero-emission" fuels, e.g., e-fuels. In HighCE, we assume a logistic increase to a 100% BEV share by 2035, driven by strong implementation of the EU regulation. LowCE assumes a weakening or delay of the regulation and a logistic increase to 90% in 2050.

For the current steel intensity of BEVs and ICEVs, we use estimates by an Öko-Institut report[10]. Historically, we assume that in the last 20 years, the steel intensity per car has risen at the same rate as the average overall weight per car, namely by 1%/year[10]. In the future, we assume that the steel intensity will remain constant in BaseCE. In HighCE, we assume that the steel intensity will decrease radically to 240 kg for BEVs and 300 kg for ICEVs in 2050[11] (around 73% and 66% reductions compared to 2023) – based on a lightweighting scenario, for instance, with aluminum and carbon fiber[12]. In LowCE, we assume a future increase in steel intensity in line with a projection of an average weight of 0.5%/year[10] until 2040 and constant thereafter.

For the historic and projected share of flat steel versus long steel, we assume 80%-20% for ICEVs and 87%-13% in BEVs[10].

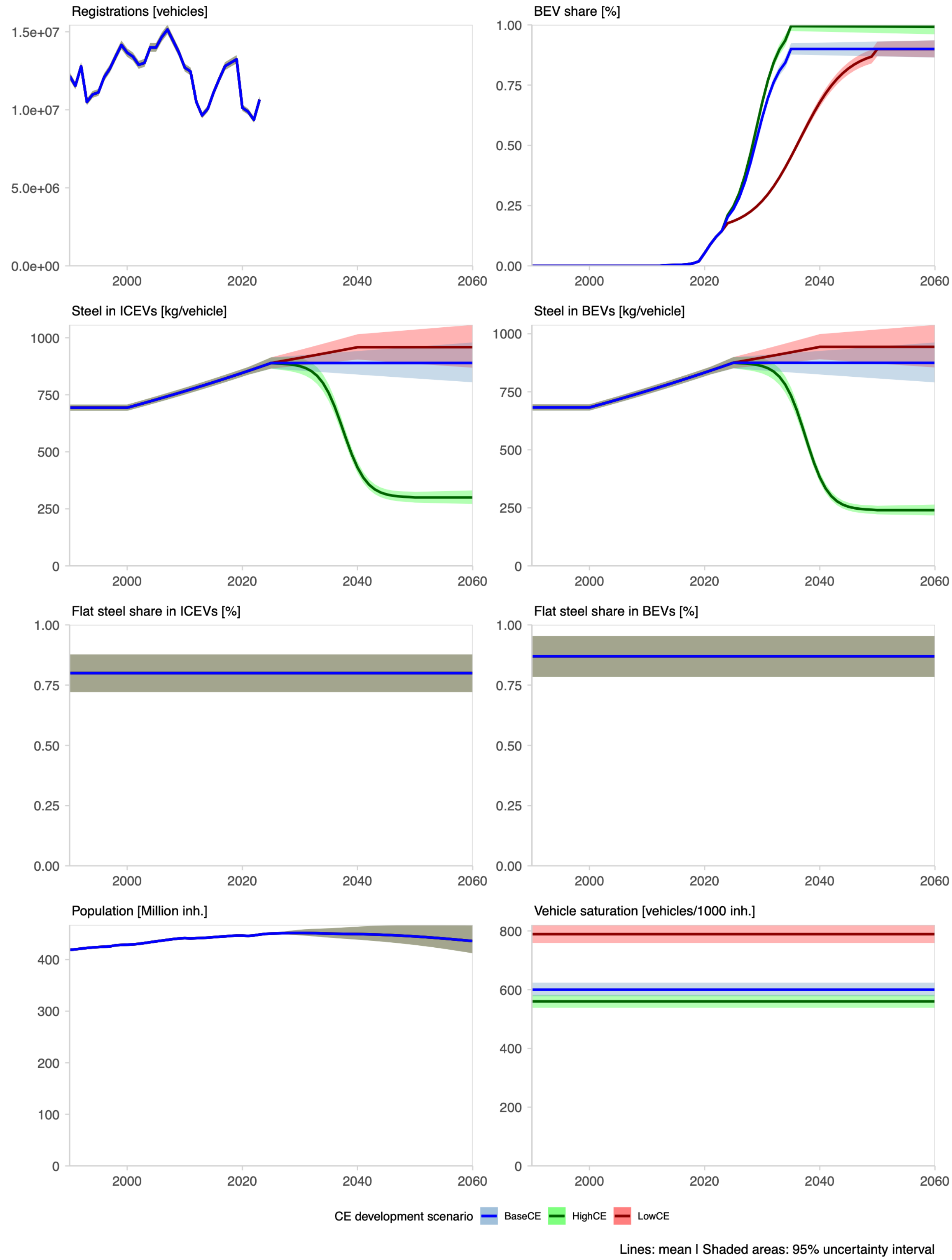

Figure 2: Input data for vehicle production.

2.2 Vehicle use phase

In the vehicle use phase, we calculate the outflows, i.e., the number of ELVs and steel entering the waste management system, using a lifetime distribution and collection losses. The input data is described below and shown in Figure 3.

For historical modeling, we apply a Weibull distribution ϕ_c with shape parameter $k = 3.6$ and scale parameter of $\lambda = 24.2$, representing an average vehicle lifetime of 21.8 years across EU27[13]. The Weibull shape parameter will remain constant across all three CE development scenarios for future age-cohorts. However, the future vehicle lifetime and associated Weibull scale parameter vary by CE development scenario. For BaseCE, we assume that the vehicle lifetime remains constant after 2021. For HighCE, new cars have higher longevity; hence, the lifetime of new age-cohorts increases logistically by 30% from 2021 to 2040. For instance, consumers use cars longer as BEVs require less maintenance, and car manufacturers may prioritize long-term customer retention through digital tools and over-the-air software updates. For LowCE, we assume the lifetime of new age-cohorts decreases logistically by 30% from 2021 to 2040. This scenario may arise due to the currently high intra-EU variation[13]: if countries with currently older cars follow the trend of countries with younger cars as their economies further develop. Higher planned obsolescence may exacerbate this trend, and, especially for BEVs, faster technological improvements may lead to more frequent car replacements.

With the information in chapter 2.1 and the Weibull distribution, we calculate the stocks, inflows, and outflows of cars and their associated steel. Car outflows are the predicted End-of-Life Vehicles (ELVs) in a year. However, not all ELVs may enter the formal waste management system within the EU, which we quantify as collection losses in PARCS. Hence, to determine the ELVs and associated steel in F_{23}, we deduct two collection losses from the outflows: extra-EU net exports (γ_t^{exp}, F_{20a}) and unknown whereabouts (γ_t^{unk}, F_{20b}).

ELVs leave the EU. We use net export losses in % for 2010 until 2014[14] and for 2019[15] on ELVs arising in the model. Before 2010, we assume the average of 2010 until 2014. For 2015-2018, we assume the average of 2014 and 2019. In all CE development scenarios, the 2019 value will remain constant (we still account for year-on-year variability with the Monte Carlo simulation). These are officially tracked exports of used cars in trade databases. Therefore, we assume they cannot be avoided and are part of regular trade patterns. Strictly speaking, extra-EU used vehicle net exports include vehicles of all ages, not only ELVs. Since their age distribution is unknown, the theoretically derived ELVs are the only meaningful basis for applying collection losses. The net export loss percentage, therefore, serves as a correction factor, capturing in aggregate the share of vehicles no longer available for formal end-of-life treatment in the EU due to prior export. Changes in illegal exports are covered in the next collection loss below.

Illegal or informal ELV treatment is widespread. These ELV losses are referred to as unknown whereabouts. We assume they do not enter the formal waste management system and therefore account for them as losses. We use losses in % for 2010 until 2014[14] and for 2019[15] on ELVs arising in the model. Before 2010, we assume the average of 2010 until 2014. For 2015-2018, we assume the average of 2014 and 2019. After 2019, in BaseCE, losses decrease logistically by twice the standard deviation of 2008-2019 levels by 2050. In HighCE, losses decrease logistically to 0% by 2050. In LowCE, losses will remain constant at the 2019 value. The rationale is that, in both BaseCE and HighCE, improved tracking systems, product passports, stricter handling of

certificates of destruction, and limits to untracked extra-EU ELV exports, as planned in the EU ELV Regulation Proposal[16], will come to fruition.

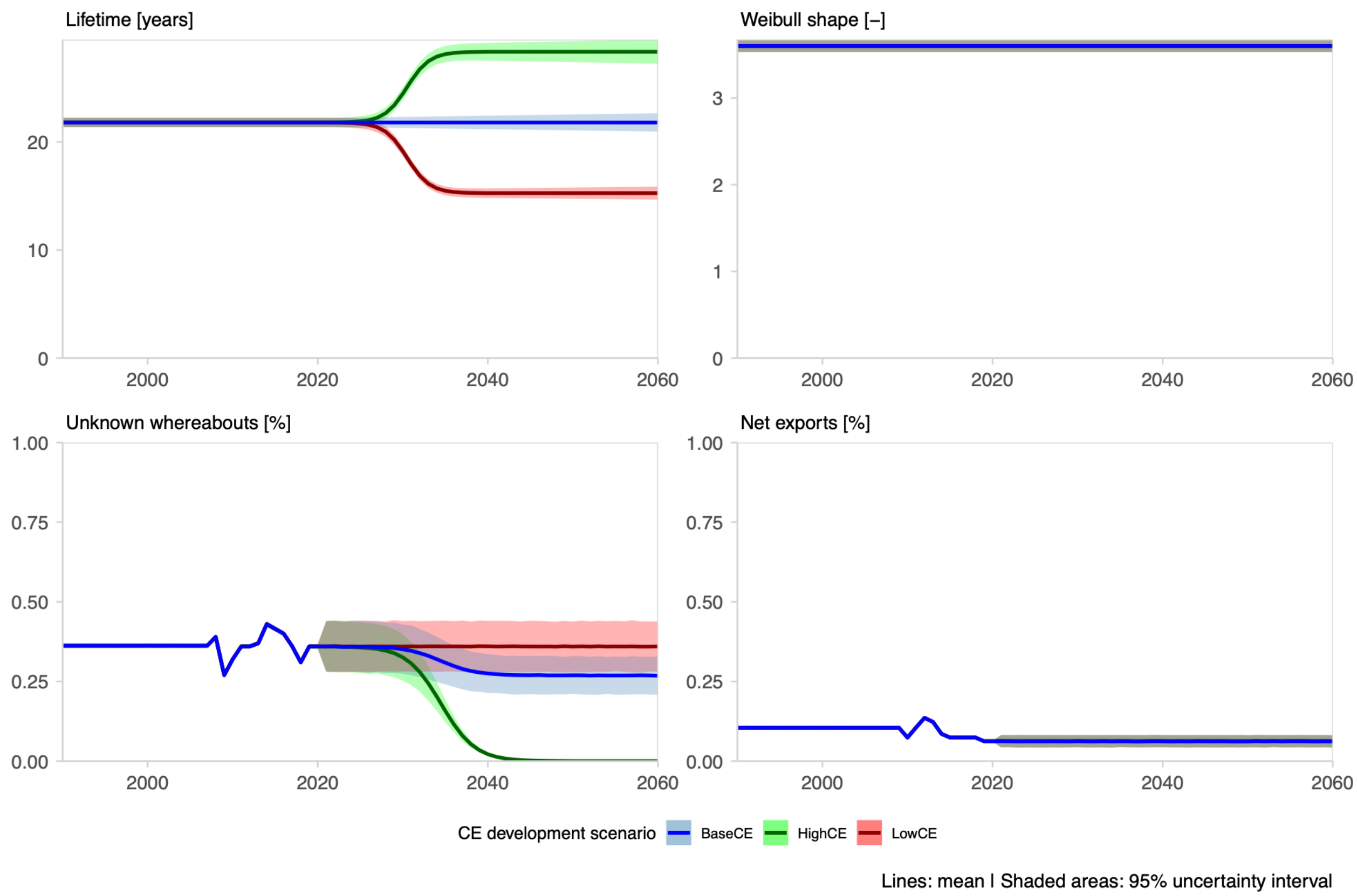

Figure 3: Input data for the vehicle use phase.

2.3 Automotive scrap allocation

We require recycling losses and a scrap allocation logic in the automotive scrap allocation process. The assumptions are below, and the input data is in Figure 4.

To calculate the available scrap, we deduct the recycling losses σ. Steel recycling has been practiced for a long time, and recycling losses F_{30} are low. We assume a constant recycling efficiency $\sigma = 97\%$ based on two sources[15,17], both historical and future.

The automotive scrap allocation process defines how the steel scrap is allocated. Closed-loop flat steel for automotive applications has higher quality requirements compared to automotive long steel or building steel. Assuming that the steel scrap meets the requirements for closed-loop flat steel, the following hierarchy logic is implemented: We assign flows to (1) closed-loop flat steel production, (2) closed-loop long steel production, and (3) buildings, subject to a maximum technical potential.

Quality constraints limit scrap use, so we define a maximum post-consumer scrap use rate per allocation route, i.e., the scrap use rate per unit of new steel product (before remelting and fabrication losses). Automotive flat steel has the highest quality requirements, and tramp elements like copper often contaminate scrap. Hence, the maximum post-consumer scrap use rate for flat steel production is currently $\omega_t^{max,\,FS} = 0\%$[10]. For long steel, we assume $\omega_{t\leq2025}^{max,\,LS} = 33\%$ until

2025[10]. In BaseCE, an increase to 90% is possible[10], which can be reached gradually by 2050. In HighCE, we assume a faster improvement to 90% by 2040. The values of 33% and 90% refer to the recycled content potential, including pre-consumer scrap, according to Sutter et al. Here, we assume the same percentages can be achieved solely from post-consumer waste, as we expect improvements in waste management practices, and the quality requirements for long steel are not as high as for flat steel. In LowCE, we assume no improvement. F_{34} represents the flow of automotive scrap going into automotive flat steel and long steel production.

Scrap that cannot be used for automotive flat steel and long steel production goes to the buildings sector in F_{38}. For this flow, the buildings sector does not have a maximum post-consumer scrap use rate; we assume that all automotive scrap is of sufficient quality for use in the buildings sector. The academic literature discusses this "downcycling" trend extensively, in the case of steel, coined "car to rebar," with the latter being a widespread steel product in buildings[18].

Currently, the maximum post-consumer scrap use rate for automotive flat steel production is 0%[10,19]. Without dedicated efforts, this value will not change – hence, it remains constant at 0% in all three CE development scenarios. In case of a dedicated push by policymakers to mandate or by the industry to pursue higher closed-loop circularity levels, the maximum post-consumer scrap use rate for automotive flat steel production must improve. We discuss options to increase this rate in Section 4, where we model a potential improvement as a separate sensitivity analysis.

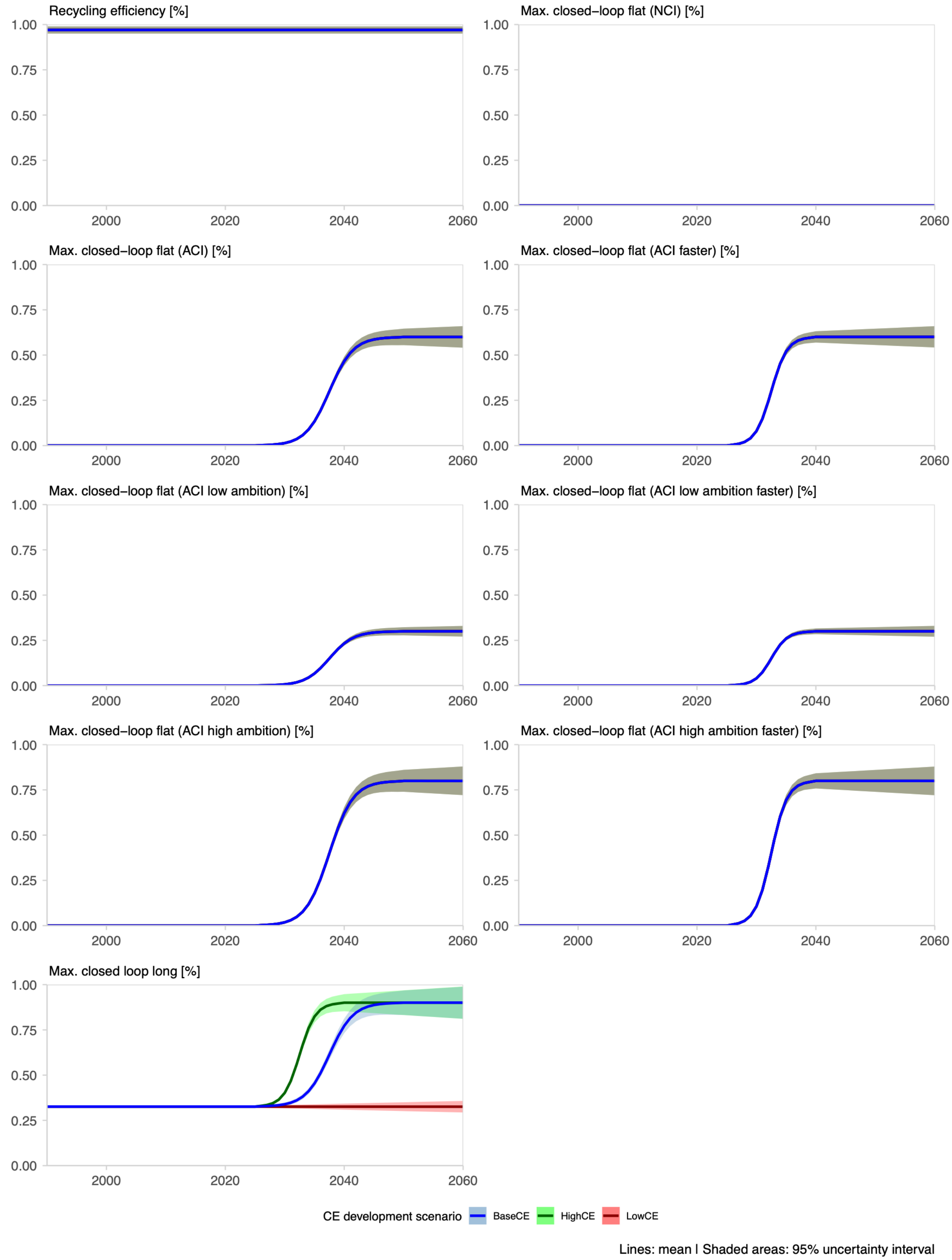

Figure 4: Input data for automotive scrap allocation.

2.4 Automotive steel production

Scrap must be processed into the final steel, ending up in new cars, as a proportion of F_{41}. First, scrap undergoes remelting losses during steel production. We assume the losses are the same for long steel and flat steel, i.e., $\rho^{LS} = \rho^{FS} = \rho$, based on the assumption that the Electric Arc Furnace (EAF) route and Blast Furnace-Basic Oxygen Furnace (BF-BOF) have similar remelting losses. We approximate the remelting losses based on multiple sources[20–22] as $\rho = 12\%$. This value is the same across all three CE development scenarios, assuming steel production is established, and the room for efficiency improvements is limited.

The BF-BOF route is typically used for high-quality steel, with virgin iron and up to 20% high-quality scrap added to the converter as a coolant. Currently, this is high-quality scrap (prompt or new scrap) from manufacturing processes (i.e., pre-consumer waste or reutilization flows[ii]) – not post-consumer scrap. The EAF route can be fully scrap-based or combine scrap with Direct Reduced Iron (DRI). The scrap use rate can be close to 100% in an EAF, but primarily for low-quality applications in the building sector, or partially for long steel products in the automotive industry. To produce flat steel for high-quality applications, such as in the automotive sector, the scrap quality must be high, and dilution with DRI is required. To reduce model complexity, we do not explicitly model the EAF/DRI and BF-BOF share, as this is implicit in the maximum scrap-use rate. Section 4 discusses the implications of this decision.

After remelting, the final step is fabrication. Fabrication losses (λ_t^p, F_{10}) arise when converting steel into the final product during manufacturing, i.e., a car. For long steel, we use a value of $\lambda^{LS} = 10\%$ based on our industry expertise. Again, we use the same value across all three CE development scenarios, assuming the fabrication process is established and there is limited room for efficiency improvements. For flat steel, however, the potential for improvement is higher. Based on the study by Horton & Allwood[23], current fabrication losses for sheet metal in the automotive sector are around $\lambda_{t\leq 2017}^{FS} = 44\%$. According to the researchers, improved manufacturing could reduce fabrication losses to 30%. In BaseCE, we assume a logistic decrease to 30% by 2050. We consider an improvement even in BaseCE because there is a cost incentive for manufacturers to reduce losses. In HighCE, we make faster progress toward material efficiency and assume a logistic decrease to 30% by 2040. In LowCE, we presume the fabrication losses remain constant.

[ii] See Section 6 “Definitions of scrap” for details on scrap terminology.

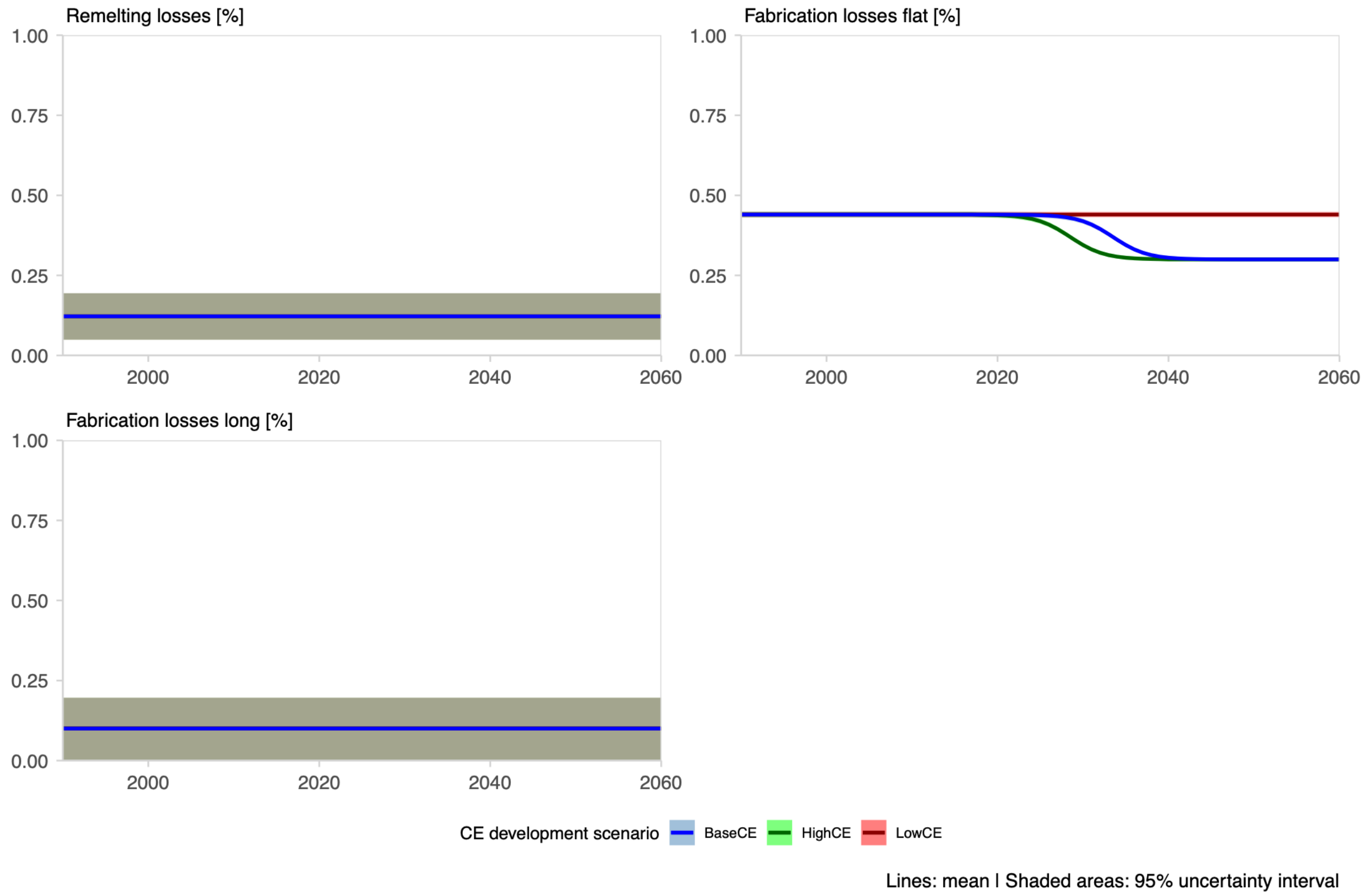

Figure 5: Input data for automotive steel production.

3 Modeling the building sector

Figure 6 shows the building sector, including all the processes, stocks, and flows. In the following, we describe the assumptions and data inputs for processes 5, 6, 7, and 8.

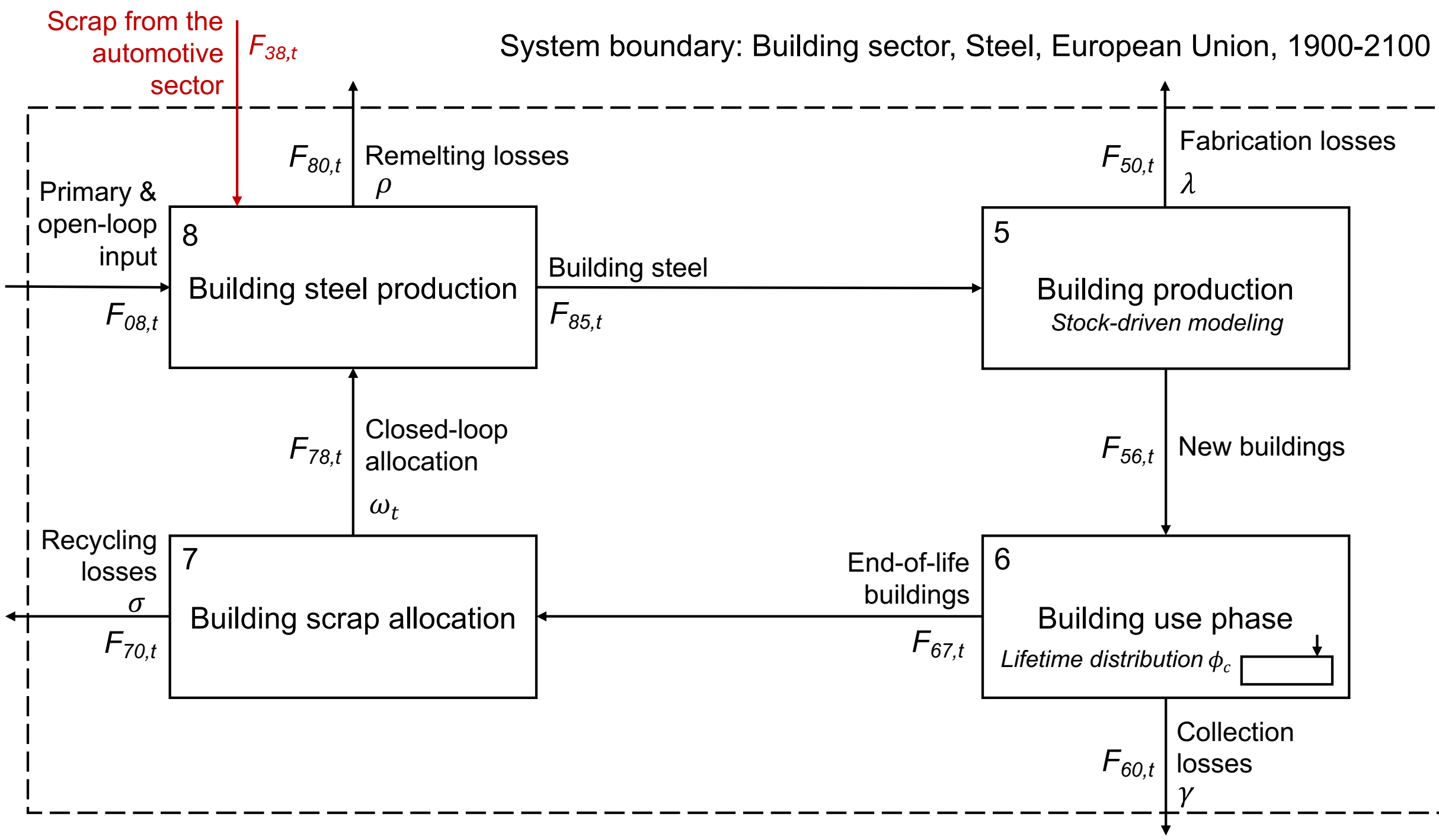

Figure 6: Building sector in PARCS.

3.1 Building production

We use floor space (m^2) as the unit for buildings. We calculate the building and associated steel inflows F_{56} into the building use phase based on the current building stock in 2020 (including age distribution), the projected future stock, and the historic and projected steel intensity per building, differentiated by residential and nonresidential buildings. Figure 7 shows the input data.

For the historical simulation, we use country-level building stock statistics from the EU Building Stock Observatory[24] and sum them up for the EU27. The dataset provides the building stock in 2020 and the age-cohort distribution, i.e., how much floor space is provided by buildings built before 1945, between 1946 and 1959, and so forth. Thanks to this age-cohort information and our assumed lifetime distribution (section 3.2), we can "hindcast" the historic stocks and flows using stock-driven modeling.

We also perform stock-driven modeling for future simulations from 2021 to 2100. Based on the known building stock per inhabitant in 2020, we project how the building stock per inhabitant will evolve and multiply by the projected population according to Eurostat[7]. In BaseCE, we assume the building stock per inhabitant remains constant at the 2020 level until 2100. In HighCE, we assume the building stock per inhabitant will decrease linearly by 25% from 2020 to 2100. In LowCE, we assume the building stock per inhabitant will increase linearly by 20% from 2020 to 2100. We base our assumption on a study by Pauliuk et al.[25] that includes projections with a similar range. The rationale is that, in the case of high CE efforts, society will cope with less floor space per person in the long run, e.g., by building more apartments and high-rise buildings. The opposite trend holds for low CE efforts.

To calculate the steel inflows F_{56} into the building use phase, we multiply the historic building stock by the historic steel intensity factors in kg/m^2 provided by Lotz et al.[26]. For the future steel

inflows, we use projections of how steel intensity will evolve. In BaseCE, we assume it remains constant until 2100. In HighCE, we assume that steel intensity will decrease logistically by 3% from 2021 to 2100, thanks to more efficient building practices and alternative materials. In LowCE, we assume the opposite: steel intensity will increase logistically by 3% from 2021 to 2100. We base these projections on a study with similar ranges[25].

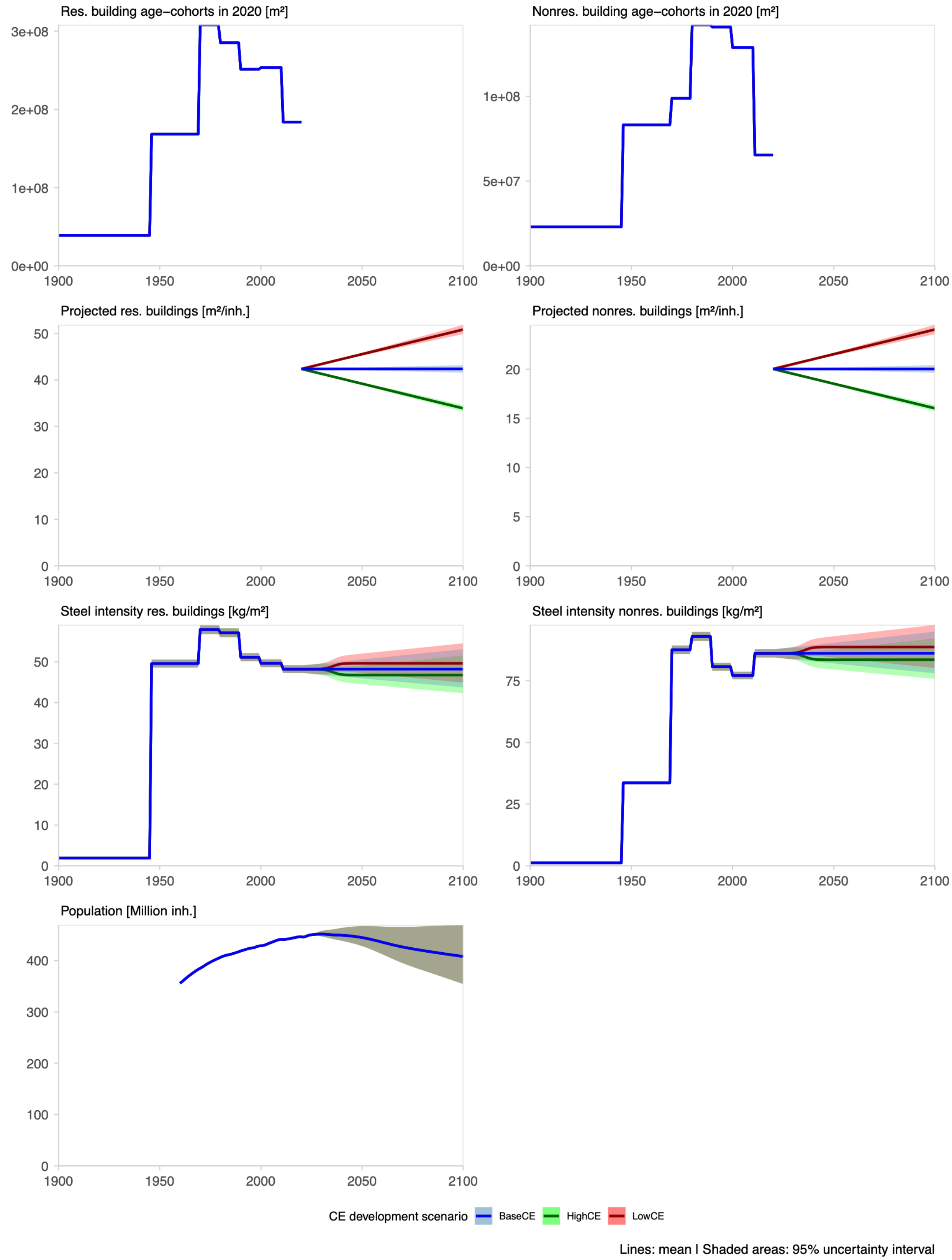

Figure 7: Input data for building production.

3.2 Building use phase

For the historic modeling, we apply a Weibull distribution ϕ_c with the shape parameter $k = 3$, according to Böcher et al.[27]. The same authors also report an average building lifetime of 223 years for buildings older than 1945 (Weibull scale parameter $\lambda = 240$) and an average lifetime of 89 years for buildings constructed after 1945 (Weibull scale parameter $\lambda = 100$)[27].

We hold the shape parameter constant for future simulations in all three CE development scenarios. The average building lifetime remains constant at 89 years in BaseCE and LowCE. In HighCE, however, we assume new building practices enable a 90% improvement of the expected lifetime[25], which we model with a logistic increase until 2050.

As collection losses, we assume a $\gamma = 10\%$[22] in all CE development scenarios and years.

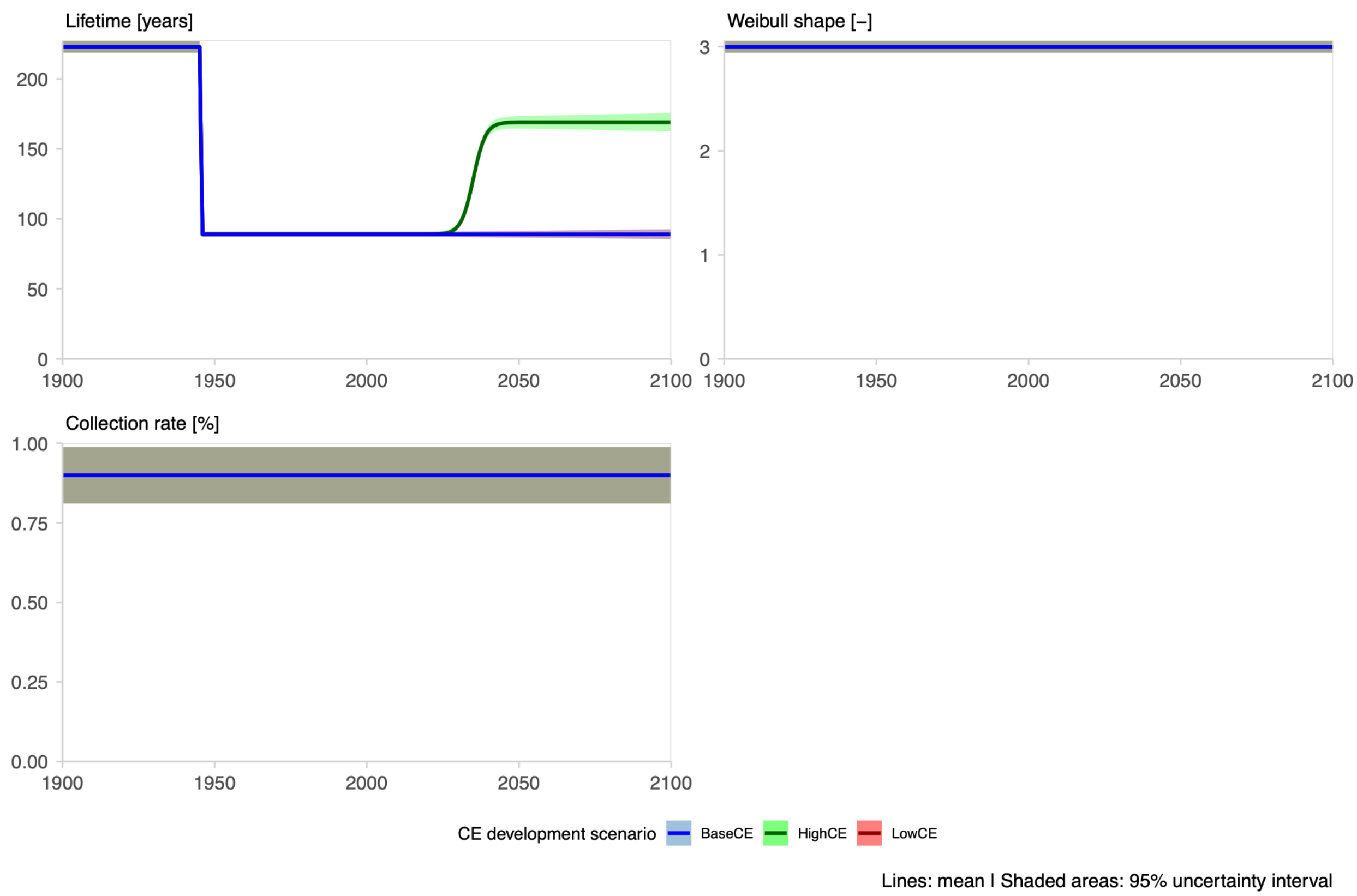

Figure 8: Input data for the building use phase.

3.3 Building scrap allocation

To calculate the available scrap, we deduct the recycling losses σ_t. Here, the logic differs from that in the automotive sector. Instead of defining a maximum scrap-use rate, we account for quality loss differently. We set historic recycling losses of 15%[22], which are higher than the 3% for ELVs, but no constrained maximum post-consumer scrap use rate as is in the automotive sector. This choice follows the idea that not all steel scrap from end-of-life buildings can be kept in a closed-loop. We assume the value decreases to 10% by 2050 in BaseCE and LowCE, and 5% by 2050 in HighCE, based on similar ranges provided by Pauliuk et al.[22]. We assume that all the remaining available scrap is allocated to the building sector (ω_t, F_{78}) as it has the lowest quality requirements.

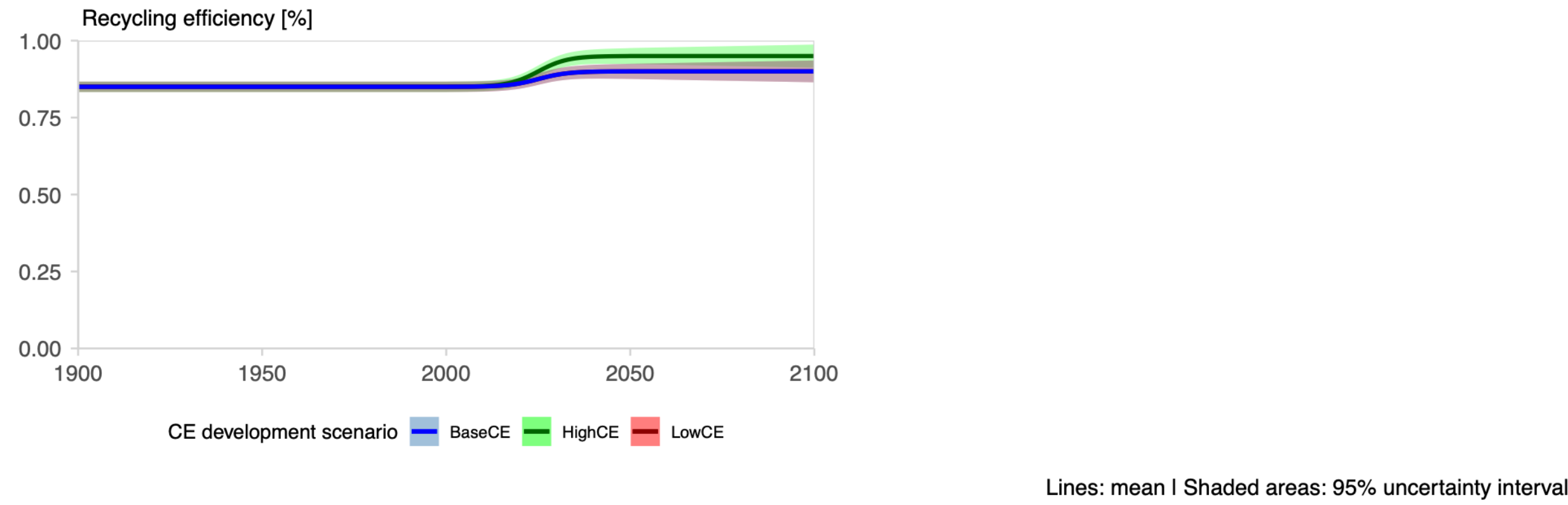

Figure 9: Input data for the building scrap allocation.

3.4 Building steel production

The scrap from the closed-loop allocation F_{78} and the scrap diverted from the automotive sector F_{38} must be processed into final steel in new buildings. We approximate the remelting losses based on three sources[20–22] as $\rho = 12\%$. This value is the same across all three CE development scenarios and over time, assuming that steel production is established and the room for efficiency improvements is limited. The same thinking applies to the fabrication losses, which we approximate as $\lambda = 7\%$ based on two sources[22,28].

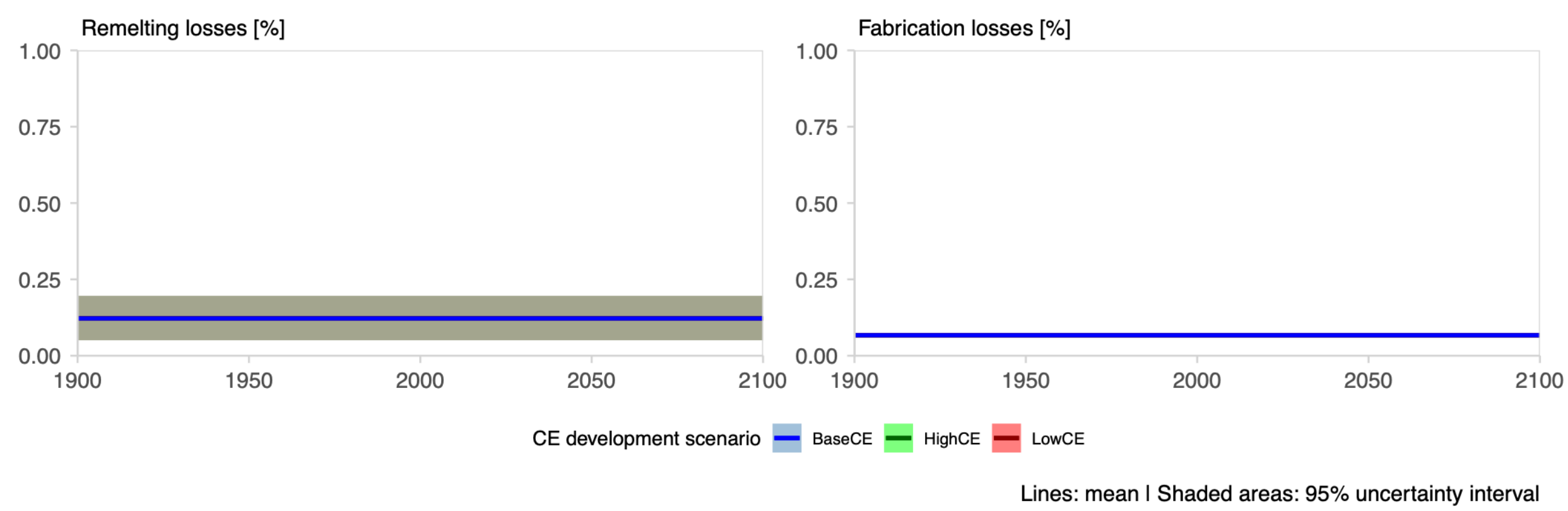

Figure 10: Input data for the building steel production.

4 Modeling an automotive closed-loop circularity intervention

As mentioned in chapter 2.3, the automotive scrap allocation logic is at the heart of PARCS and this study. Currently, virtually no post-consumer scrap from ELVs can be used to produce flat steel of sufficient quality for new vehicles[10]. Flat steel typically accounts for more than 80% of steel in new cars. Hence, increasing the maximum post-consumer scrap use rate for flat steel is crucial to improving closed-loop circularity in the automotive sector. Achieving this assumes that scrap quality improves. Given the high sensitivity of this assumption, we do not model this improvement within individual CE development scenarios. Efforts to improve the quality of scrap can occur across all three CE development scenarios. Thus, we perform a separate sensitivity analysis.

Steel scrap gets increasingly contaminated with so-called tramp elements, mostly copper[29]. If the copper concentration in scrap is too high, it cannot be used for high-quality applications like flat steel in the automotive sector. Particularly for steel scrap from ELVs, there are growing challenges related to copper contamination as vehicle electrification advances[19]. Today, most scrap is diverted to buildings and construction with lower quality requirements[22]. Daehn et al.[30] highlight the resulting risk of growing unusable steel scrap. There is potential to improve scrap quality, for instance, through part-based sorting and alloy management, as shown in a previous study of ELVs in Japan[31]. Achieving automotive closed-loop circularity may require multiple interventions along the supply chain, such as increased disassembly, improved shredding and sorting, chemical extraction, increased copper tolerance, and reduced copper content[30].

A recent study by Gross & Hermine (2025)[19] describes an interesting trial on ELVs in France. Thanks to improved dismantling techniques before shredding, namely manual wiring removal, the copper content in ELV steel scrap was reduced to 0.09% - compared to the current 0.4%. This concentration is below the threshold of 0.15% required for automotive flat steel production[19]. Currently, up to 60% of high-quality post-consumer scrap can be used for flat steel production in the United States. We assume that the same holds within the EU, i.e., a potential maximum closed-loop scrap use rate of 60% for closed-loop automotive flat steel production. Initially, the BF-BOF route could use higher-quality scrap. The BF-BOF route is predominant in the EU and can accept up to 20% high-quality scrap as coolant (currently covered with prompt scrap). In the future, reaching a maximum post-consumer scrap use rate of 60% may require upgrading EAF routes to accept more scrap[32] and sufficient availability of DRI[19]. We assume this improvement will follow a logistic (S-curve) increase from 0% to 60% by 2050. Another crucial assumption is that the 60% value achieved in a trial of a waste stream consisting solely of ELVs can be replicated at larger scales within the waste management system. This could be the case if ELVs are processed separately on a larger scale, too, or if the sorting and processing of mixed scrap streams improve over time.

It is worth noting that the model does not explicitly track quality changes in the scrap diverted to the building sector under the ACI case. The quality implications depend on the intervention: if scrap quality is improved through deeper dismantling before shredding – as modeled here – copper is removed at source, potentially benefiting the quality of scrap available to both sectors. If quality improvements were instead achieved through post-shredder technology, selectively diverting cleaner fractions to the automotive sector, the residual available to buildings could be of lower quality than before, implying higher recycling losses than modeled. In that case, the building sector circularity potential modeled here would constitute an upper bound.

The cases used in the manuscript are the *no closed-loop intervention* (NCI) case, assuming a maximum post-consumer scrap use rate of 0%, and the *automotive closed-loop intervention* (ACI) case, assuming a maximum post-consumer scrap use rate increasing from 0% to 60% by 2050 along a logistic S-curve. We further test the sensitivity of the ACI case with five more options: *ACI faster*, assuming a faster increase to 60% by 2040, *ACI low ambition*, assuming an increase to 30% by 2050, *ACI low ambition faster* with 30% by 2040, *ACI high ambition*, assuming an increase to 80% by 2050, and *ACI high ambition faster* with 80% by 2040. The results of the ACI variations are in Figure 13.

5 Quantifying uncertainties with a Monte Carlo simulation

The MC simulation approach in PARCS assumes a deterministic trend over a defined time horizon and produces simulation runs as output. PARCS differentiates between (i) fixed values, (ii) bounded values (0–1), (iii) positive unbounded quantities, and (iv) variables with smooth vs. volatile uncertainty.

Distributions:

Lognormal (mean-preserving): Applied to unbounded, positive variables (e.g., population, steel intensity).

Normal: Applied multiplicatively around one and truncated to [0,1] for bounded variables (e.g., shares, efficiencies).

None: Applied to fixed parameters when all MC simulations equal the deterministic trend.

Draw modes:

Single: Applies one random shock per path consistently across all years. Produces smooth "fan" structures.

Per year: Applies independent shocks per year and produces year-to-year variability, suitable for volatile series (e.g., exports, unknown whereabouts).

Uncertainty over time:

History: A constant historical standard deviation σ_{hist} scales the trend.

Future: uncertainty is parameterized by a standard deviation, either constant ($\sigma_{start} = \sigma_{end}$) or increasing linearly from an initial to a final standard deviation ($\sigma_{start} \leq \sigma_{end}$). The latter leads to a gradual widening of uncertainty bands.

Default: $\sigma_{hist} = \sigma_{start}$ to avoid a sudden jump from historic to future uncertainty.

For each variable, we define the distribution and the draw mode, select if a historic MC simulation is desired (if so, provide the last deterministic historic year and σ_{hist}), indicate σ_{end} and σ_{start} (defaults to σ_{hist}), and indicate if a truncation to [0,1] is required.

6 Definitions of scrap

Figure 11 provides a stylized representation of scrap flows. The focus in PARCS is on post-consumer waste.

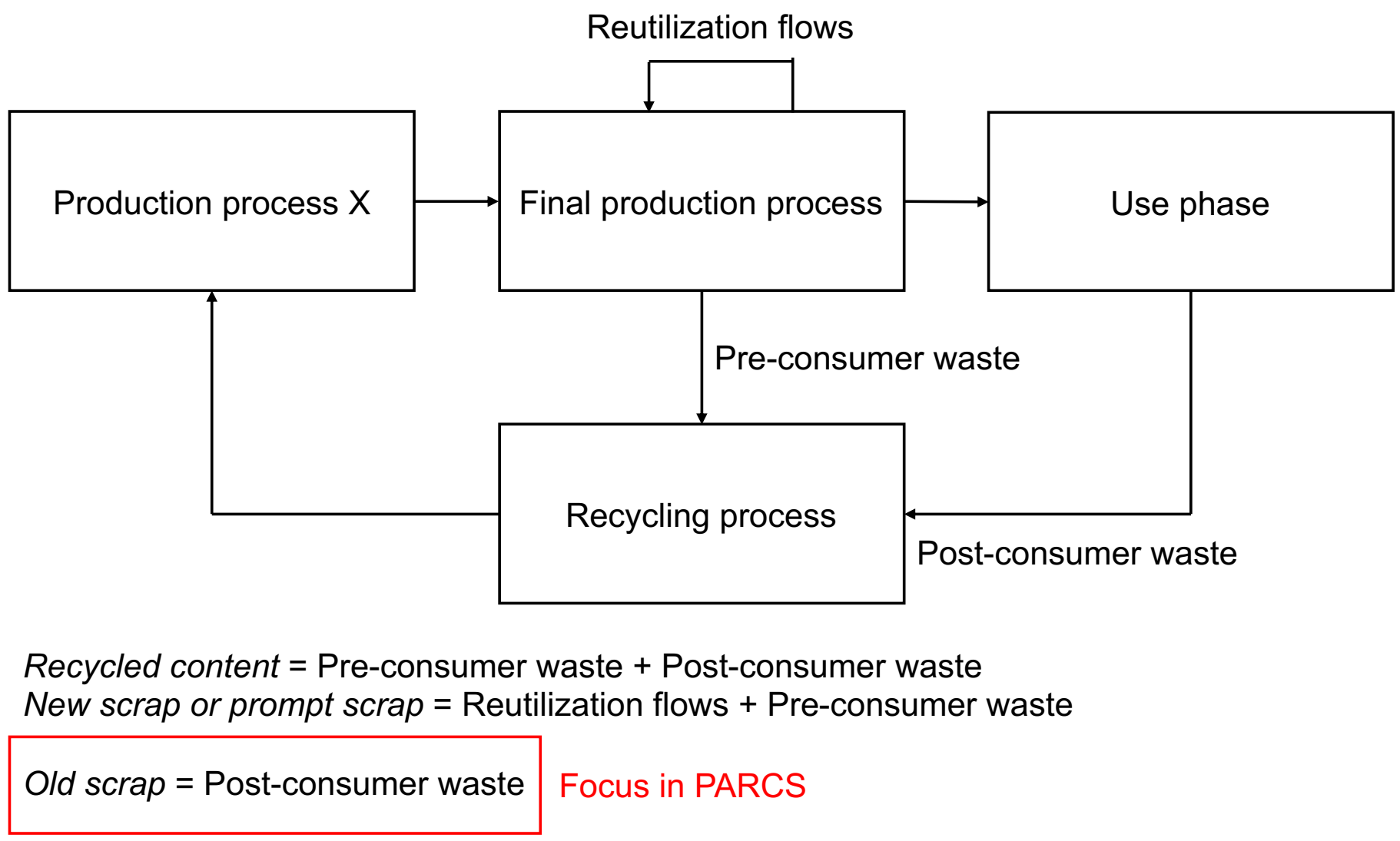

Figure 11: Stylized representation of scrap flows.

7 Additional results

We assess circularity in the automotive and building sectors using the CLOP and $CLOP^{PLUS}$ indicators, including MC uncertainty bands, at the "gross" level as shown in Figure 12. "Gross" refers to an alternative CLOP where both recycled content supply and steel demand are defined at the "gross" level, i.e., before remelting and fabrication losses.

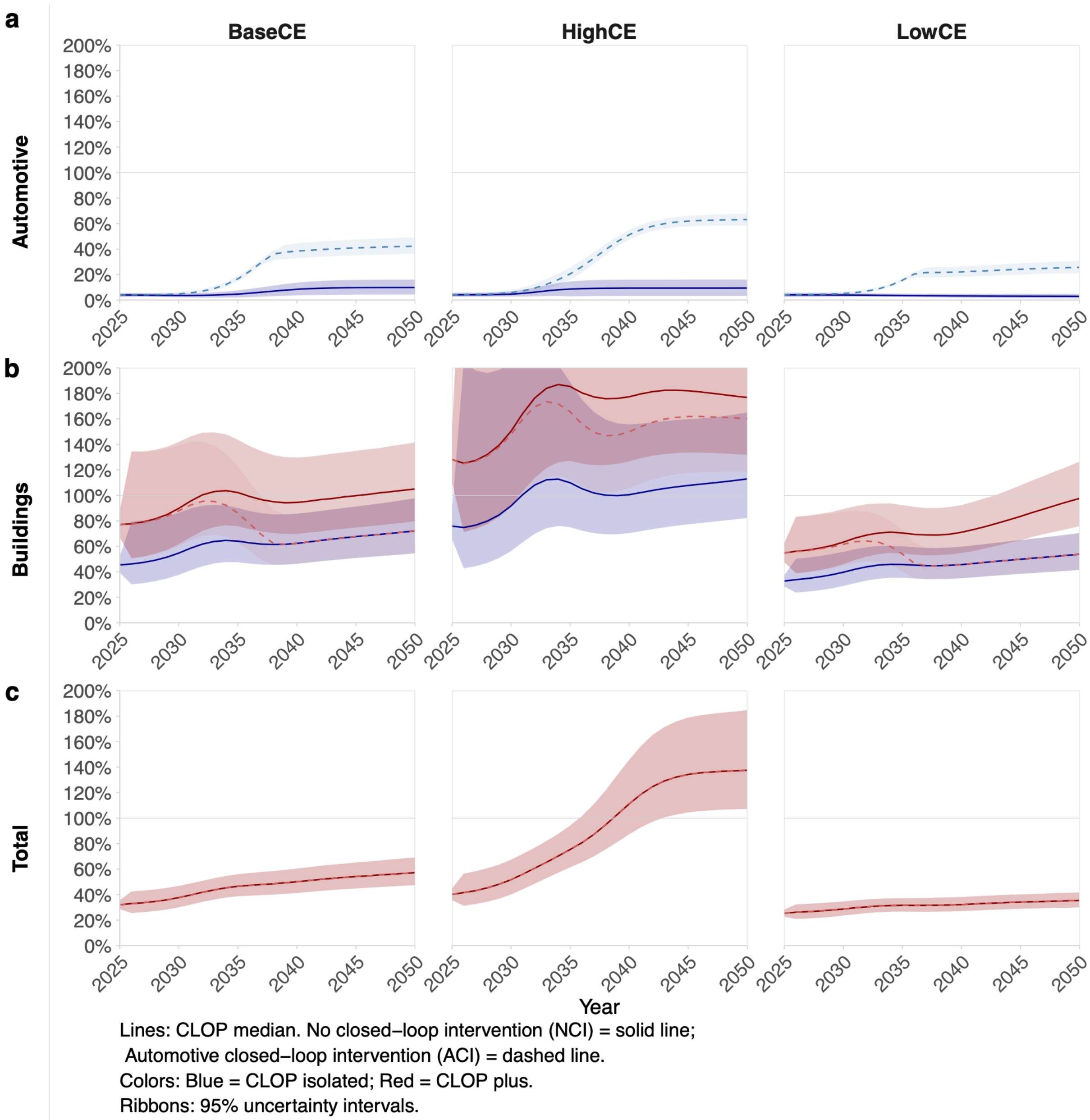

Figure 12: The Closed-Loop Post-Consumer Circularity Potential (CLOP) at the "gross" level for three CE development scenarios (BaseCE, HighCE, and LowCE) and three perspectives (a) Automotive, (b) Buildings, and (c) Total, where "Total" is the combination of the automotive and building sectors. "Plus" in the building industry includes the scrap diversion from the automotive industry. "Gross" refers to an alternative CLOP where both recycled content supply and steel demand are defined at the "gross" level, i.e., before remelting and fabrication losses. A value higher than 100% means that more scrap would be available from post-consumer sources than is necessary to cover steel demand in the EU.

We present the sensitivity analysis of CLOP and CLOP^{PLUS} to the ACI cases in Figure 13. Two observations stand out: First, in BaseCE and LowCE in the automotive sector, CLOP is below the maximum post-consumer scrap use rate and is therefore constrained by scrap availability. In HighCE, scrap availability is sufficient for CLOP to match the maximum post-consumer scrap use rate. Second, in the buildings sector, CLOP^{PLUS} shows patterns in the ACI sensitivity cases similar to those in the original ACI case. There are expected variations due to the underlying assumptions

regarding the level and timing of the maximum post-consumer scrap use rate in each ACI sensitivity.

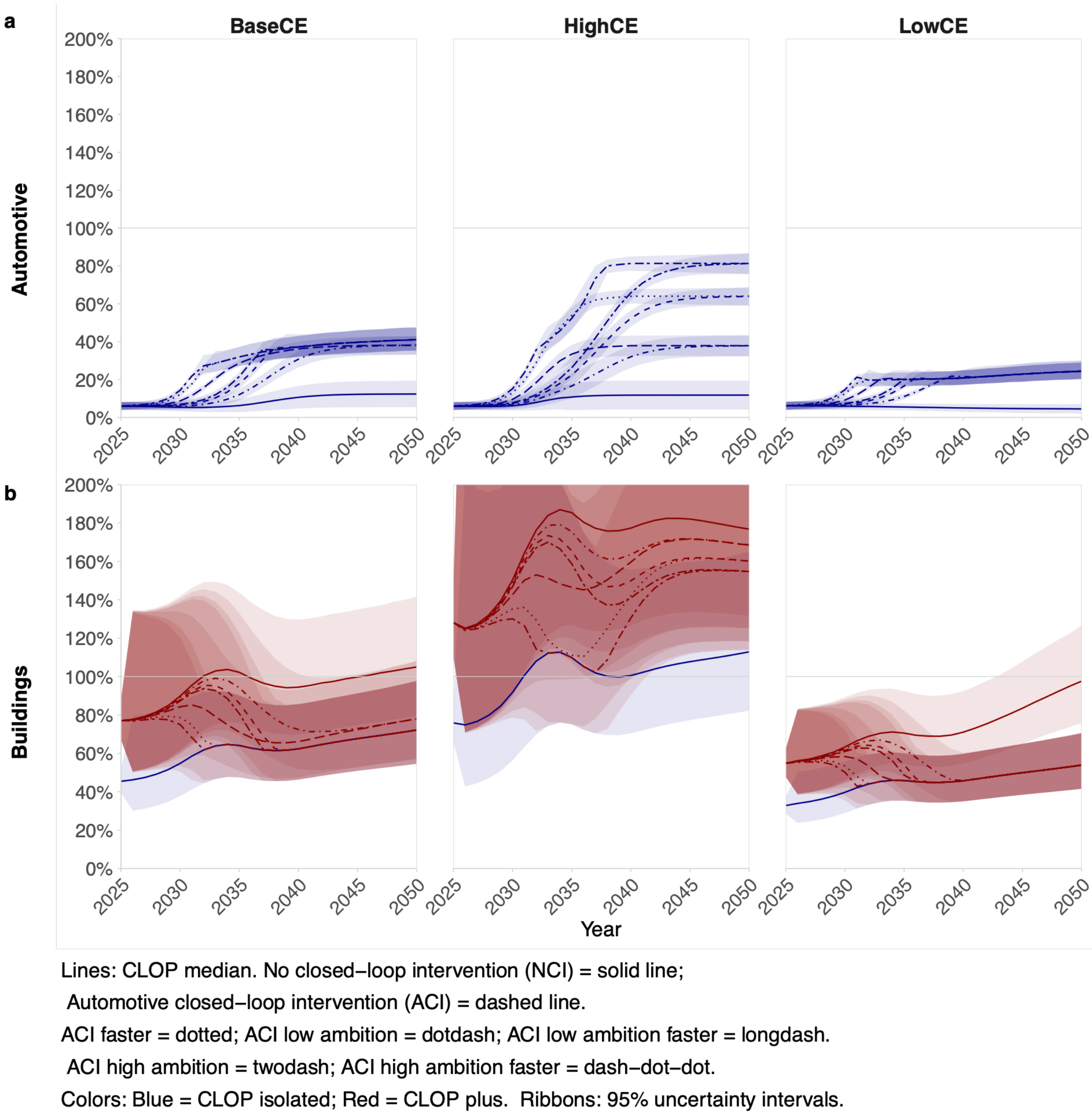

Figure 13: The Closed-Loop Post-Consumer Circularity Potential (CLOP) for three CE development scenarios (BaseCE, HighCE, and LowCE) and two perspectives (a) Automotive, (b) Buildings. It also includes a sensitivity analysis of the Automotive closed-loop intervention case (ACI), which includes the variations "ACI faster", "ACI low ambition", "ACI low ambition faster", "ACI high ambition", and "ACI high ambition faster." "Plus" in the building industry includes the scrap diversion from the automotive industry. A value higher than 100% means that more scrap would be available from post-consumer sources than is necessary to cover steel demand in the EU.

References

(1) Wiedenhofer, D.; Fishman, T.; Lauk, C.; Haas, W.; Krausmann, F. Integrating Material Stock Dynamics Into Economy-Wide Material Flow Accounting: Concepts, Modelling, and Global Application for 1900–2050. *Ecological Economics* **2019**, *156*, 121–133. https://doi.org/10.1016/j.ecolecon.2018.09.010.

(2) Reichert, D.; Maeder, M.; Fröhling, M. Assessing and Calculating Closed-Loop Recycled Content: The Product-Oriented Closed-Loop Material Indicator (PCMI). *Resour. Conserv. Recycl.* **2025**, *219*, 108266. https://doi.org/10.1016/j.resconrec.2025.108266.

(3) Eurostat. *New passenger cars by type of motor energy and engine size*. Data Browser. https://doi.org/10.2908/ROAD_EQR_CARMOT.

(4) Eurostat. *Population change - Demographic balance and crude rates at national level*. Data Browser. https://doi.org/10.2908/DEMO_GIND.

(5) Eurostat. *Passenger cars - per thousand inhabitants*. Data Browser. https://doi.org/10.2908/ROAD_EQS_CARHAB.

(6) Zhou, H.; Yang, Y.; Li, W.; McKechnie, J.; Thiede, S.; Wang, P. EU's Recycled Content Targets of Lithium-Ion Batteries Are Likely to Compromise Critical Metal Circularity. *One Earth* **2024**, *7* (7), 1288–1300. https://doi.org/10.1016/j.oneear.2024.06.017.

(7) Eurostat. *Population on 1st January by age, sex and type of projection*. Data Browser. https://doi.org/10.2908/PROJ_23NP.

(8) Eurostat. *New passenger cars by type of motor energy*. Data Browser. https://doi.org/10.2908/ROAD_EQR_CARPDA.

(9) The European Parliament and the Council of the European Union. *REGULATION (EU) 2023/851 OF THE EUROPEAN PARLIAMENT AND OF THE COUNCIL of 19 April 2023 Amending Regulation (EU) 2019/631 as Regards Strengthening the CO2 Emission Performance Standards for New Passenger Cars and New Light Commercial Vehicles in Line with the Union's Increased Climate Ambition*; European Union, 2023; pp 1–16. http://data.europa.eu/eli/reg/2023/851/oj (accessed 2025-09-23).

(10) Sutter, J.; Adjei, F.; Baron, Y.; Kosińska-Terrade, I. *Boosting the Use of Recycled Steel in the EU Automotive Industry under the ELV Regulation*; Darmstadt, 2025. https://www.oeko.de/fileadmin/oekodoc/ELV_Report_European-Commission_Oeko-Institut.pdf (accessed 2025-06-19).

(11) Hill, N.; Scammell, H.; Raugei, M.; Kilstein, A.; King, A. *The Use of Green Steel in the Automotive Industry*; Oxfordshire, UK, 2024. https://www.transportenvironment.org/articles/cleaning-up-steel-in-cars-why-and-how (accessed 2025-09-23).

(12) Modaresi, R.; Pauliuk, S.; Løvik, A. N.; Müller, D. B. Global Carbon Benefits of Material Substitution in Passenger Cars until 2050 and the Impact on the Steel and Aluminum Industries. *Environ. Sci. Technol.* **2014**, *48* (18), 10776–10784. https://doi.org/10.1021/es502930w.

(13) Held, M.; Rosat, N.; Georges, G.; Pengg, H.; Boulouchos, K. Lifespans of Passenger Cars in Europe: Empirical Modelling of Fleet Turnover Dynamics. *European Transport Research Review* **2021**, *13* (1), 9. https://doi.org/10.1186/s12544-020-00464-0.

(14) Mehlhart, G.; Kosińska, I.; Baron, Y.; Hermann, A. *Assessment of the Implementation of Directive 2000/53/EU on End-of-Life Vehicles (the ELV Directive) with Emphasis on the End of Life Vehicles of Unknown Whereabouts*; Publications Office: Freiburg, Germany, 2017. https://ec.europa.eu/environment/pdf/waste/elv/ELV_report.pdf (accessed 2025-09-23).

(15) Baron, Y.; Kosińska-Terrade, I.; Loew, C.; Köhler, A.; Moch, K.; Sutter, J.; Graulich, K.; Adjei, F.; Mehlhart, G. *Study to Support the Impact Assessment for the Review of Directive 2000/53/EC on End-of-Life Vehicles*; Luxembourg, 2023. https://doi.org/10.2779/855228.

(16) European Commission. *Proposal for a Regulation of the European Parliament and of the Council on Circularity Requirements for Vehicle Design and on Management of End-of-Life Vehicles, Amending Regulations (EU) 2018/858 and 2019/1020 and Repealing Directives 2000/53/EC and 2005/64/EC (Text with EEA Relevance)*; Brussels, 2023; pp 1–79. https://eur-lex.europa.eu/legal-content/EN/TXT/?uri=COM:2023:451:FIN (accessed 2023-07-14).

(17) Tasala Gradin, K.; Luttropp, C.; Björklund, A. Investigating Improved Vehicle Dismantling and Fragmentation Technology. *J. Clean. Prod.* **2013**, *54*, 23–29. https://doi.org/10.1016/j.jclepro.2013.05.023.

(18) Hundt, C.; Pothen, F. European Post-Consumer Steel Scrap in 2050: A Review of Estimates and Modeling Assumptions. *Recycling* **2025**, *10* (1), 21. https://doi.org/10.3390/recycling10010021.

(19) Gross, H.; Hermine, J.-P. *Car-to-Car Steel: Potential of End-of-Life Vehicle Deep-Dismantling and Use of Copper Depolluted Steel Scrap to Decarbonize Automotive Flat Steel Production*; Paris, France, 2025.

(20) Joint Research Centre: Institute for Prospective Technological Studies; Remus, R.; Roudier, S.; Aguado-Monsonet, M. A.; Delgado Sancho, L. *Best Available Techniques (BAT) Reference Document for Iron and Steel Production – Industrial Emissions Directive 2010/75/EU – Integrated Pollution Prevention and Control*; Publications Office, 2013. https://doi.org/10.2791/97469.

(21) Rostek, L.; Lotz, M. T.; Wittig, S.; Herbst, A.; Loibl, A.; Espinoza, L. T. *A Dynamic Material Flow Model for the European Steel Cycle*; No. S 07/2022; Karlsruhe, Germany, 2022. https://doi.org/10.24406/publica-58.

(22) Pauliuk, S.; Kondo, Y.; Nakamura, S.; Nakajima, K. Regional Distribution and Losses of End-of-Life Steel throughout Multiple Product Life Cycles—Insights from the Global Multiregional MaTrace Model. *Resour. Conserv. Recycl.* **2017**, *116*, 84–93. https://doi.org/10.1016/J.RESCONREC.2016.09.029.

(23) Horton, P. M.; Allwood, J. M. Yield Improvement Opportunities for Manufacturing Automotive Sheet Metal Components. *J. Mater. Process. Technol.* **2017**, *249*, 78–88. https://doi.org/10.1016/j.jmatprotec.2017.05.037.

(24) European Commission. *EU Building Stock Observatory*. https://energy.ec.europa.eu/topics/energy-efficiency/energy-performance-buildings/eu-building-stock-observatory_en (accessed 2025-09-22).

(25) Pauliuk, S.; Heeren, N.; Berrill, P.; Fishman, T.; Nistad, A.; Tu, Q.; Wolfram, P.; Hertwich, E. G. Global Scenarios of Resource and Emission Savings from Material Efficiency in Residential Buildings and Cars. *Nat. Commun.* **2021**, *12* (1), 5097. https://doi.org/10.1038/s41467-021-25300-4.

(26) Lotz, M. T.; Herbst, A.; Müller, A.; Kranzl, L.; Rosales Carreon, J.; Worrell, E. A Material Flow Model of Steel and Concrete in EU Buildings: National Differences of the Service-Stock-Flow Nexus. *Cleaner Waste Systems* **2024**, *8*, 100153. https://doi.org/10.1016/J.CLWAS.2024.100153.

(27) Böcher, C.; Cunha, S.; Fishman, T.; van der Voet, E.; Kippert, K.; Mogollón, J. M. A Dynamic Model to Hindcast Historical Material Stocks and Flows from Current Stocks: Application to the Buildings of 1159 Administrative Regions of the EU27, 1970–2050. *Resour. Conserv. Recycl.* **2025**, *217*, 108198. https://doi.org/10.1016/j.resconrec.2025.108198.

(28) Hatayama, H.; Daigo, I.; Matsuno, Y.; Adachi, Y. Outlook of the World Steel Cycle Based on the Stock and Flow Dynamics. *Environ. Sci. Technol.* **2010**, *44* (16), 6457–6463. https://doi.org/10.1021/es100044n.

(29) Dworak, S.; Fellner, J. Steel Scrap Generation in the EU-28 since 1946 – Sources and Composition. *Resour. Conserv. Recycl.* **2021**, *173*, 105692. https://doi.org/10.1016/j.resconrec.2021.105692.

(30) Daehn, K. E.; Cabrera Serrenho, A.; Allwood, J. M. How Will Copper Contamination Constrain Future Global Steel Recycling? *Environ. Sci. Technol.* **2017**, *51* (11), 6599–6606. https://doi.org/10.1021/acs.est.7b00997.

(31) Ohno, H.; Matsubae, K.; Nakajima, K.; Kondo, Y.; Nakamura, S.; Fukushima, Y.; Nagasaka, T. Optimal Recycling of Steel Scrap and Alloying Elements: Input-Output Based Linear Programming Method with Its Application to End-of-Life Vehicles in Japan. *Environ. Sci. Technol.* **2017**, *51* (22), 13086–13094. https://doi.org/10.1021/acs.est.7b04477.

(32) Gao, H.; Liu, J.; Daigo, I. Methodology Development for Estimating the Impact of Restriction Factors to Promote National Steel Recycling. *Resour. Conserv. Recycl.* **2025**, *215*, 108052. https://doi.org/10.1016/j.resconrec.2024.108052.
